# Integrating tuberculosis and noncommunicable diseases care in low- and middle-income countries (LMICs): A systematic review

**DOI:** 10.1371/journal.pmed.1003899

**Published:** 2022-01-18

**Authors:** Chuan De Foo, Pami Shrestha, Leiting Wang, Qianmei Du, Alberto L. García-Basteiro, Abu Saleh Abdullah, Helena Legido-Quigley

**Affiliations:** 1 Saw Swee Hock School of Public Health, National University of Singapore, Singapore; 2 Global Health Program, Duke Kunshan University, Kunshan, Jiangsu, China; 3 ISGlobal, Hospital Clínic, Universitat de Barcelona, Barcelona, Spain; 4 Centro de Investigação em Saúde de Manhiça, Mozambique; 5 School of Medicine, Boston Medical Center, Boston University, Boston, Massachusetts, United States of America; PLOS Medicine Editorial Board, UNITED STATES

## Abstract

**Background:**

Low- and middle-income countries (LMICs) are facing a combined affliction from both tuberculosis (TB) and noncommunicable diseases (NCDs), which threatens population health and further strains the already stressed health systems. Integrating services for TB and NCDs is advantageous in tackling this joint burden of diseases effectively. Therefore, this systematic review explores the mechanisms for service integration for TB and NCDs and elucidates the facilitators and barriers for implementing integrated service models in LMIC settings.

**Methods and findings:**

A systematic search was conducted in the Cochrane Library, MEDLINE, Embase, PubMed, Bibliography of Asian Studies, and the Global Index Medicus from database inception to November 4, 2021. For our search strategy, the terms “tuberculosis” AND “NCDs” (and their synonyms) AND (“delivery of healthcare, integrated” OR a range of other terms representing integration) were used. Articles were included if they were descriptions or evaluations of a management or organisational change strategy made within LMICs, which aim to increase integration between TB and NCD management at the service delivery level. We performed a comparative analysis of key themes from these studies and organised the themes based on integration of service delivery options for TB and NCD services. Subsequently, these themes were used to reconfigure and update an existing framework for integration of TB and HIV services by Legido-Quigley and colleagues, which categorises the levels of integration according to types of services and location where services were offered. Additionally, we developed themes on the facilitators and barriers facing integrated service delivery models and mapped them to the World Health Organization’s (WHO) health systems framework, which comprises the building blocks of service delivery, human resources, medical products, sustainable financing and social protection, information, and leadership and governance.

A total of 22 articles published between 2011 and 2021 were used, out of which 13 were cross-sectional studies, 3 cohort studies, 1 case–control study, 1 prospective interventional study, and 4 were mixed methods studies. The studies were conducted in 15 LMICs in Asia, Africa, and the Americas. Our synthesised framework explicates the different levels of service integration of TB and NCD services. We categorised them into 3 levels with entry into the health system based on either TB or NCDs, with level 1 integration offering only testing services for either TB or NCDs, level 2 integration offering testing and referral services to linked care, and level 3 integration providing testing and treatment services at one location. Some facilitators of integrated service include improved accessibility to integrated services, motivated and engaged providers, and low to no cost for additional services for patients. A few barriers identified were poor public awareness of the diseases leading to poor uptake of services, lack of programmatic budget and resources, and additional stress on providers due to increased workload. The limitations include the dearth of data that explores the experiences of patients and providers and evaluates programme effectiveness.

**Conclusions:**

Integration of TB and NCD services encourages the improvement of health service delivery across disease conditions and levels of care to address the combined burden of diseases in LMICs. This review not only offers recommendations for policy implementation and improvements for similar integrated programmes but also highlights the need for more high-quality TB–NCD research.

## Abstract 2

Chuan De Foo and colleagues explore mechanisms for service integration for tuberculosis and non-communicable diseases in this systematic review.

## Author summary

**Why was this study done?:**

- Low- and middle-income countries (LMICs) are facing a high prevalence of tuberculosis (TB) and noncommunicable diseases (NCDs).
- TB and NCDs share multiple risk factors and programmes that integrate both TB and NCD services that can potentially serve to tackle both TB and NCDs simultaneously.
- This study aims to provide a review of the programmes that integrate TB and NCD services in LMICs to provide recommendations to researchers and policymakers for future implementation.

**What did the researchers do and find?:**

- Through the articles searched, a framework that illustrates the different levels of integration and entry points into the health system was developed.
- There is a total of 3 levels of integration, with the deepest level of integration offering services that includes both screening and treatment services within one facility as compared to lower levels of integration that either only offers to screen or onwards referral for treatment.
- The World Health Organization’s (WHO) health systems framework was employed to map the facilitators and barriers in implementing TB and NCD integrated programmes through the dimensions of health service delivery, human resources, medical products and vaccines, sustainable financing and social protection, information, and leadership and governance.

**What do these findings mean?:**

- The findings revealed the potential applicability of programmes with the deepest levels of integration for implementation in LMICs to confer the most benefits to patients and the health system when TB and NCDs are tackled simultaneously within the same facility.
- Researchers and policymakers need to bear in mind the barriers and facilitators for the implementation of future TB and NCD integrated programmes in order to tackle both fronts concurrently and effectively.
- The dearth of evidence on TB–NCD integration has highlighted the urgency for high-quality TB–NCD research to provide robust data for formal programmatic evaluation of integrated programmes in order to provide evidence-guided lessons for future reference and deployment.

## Introduction

Tuberculosis (TB), an infectious disease derived from various species of the genus *Mycobacteria*, remains a global threat to public health [1]. Although preventable and treatable, with 1.5 million deaths every year, TB is still on the world’s top list of infectious killer diseases [1]. Furthermore, with the advent of multidrug-resistant tuberculosis (MDR-TB), first-line drugs have been rendered ineffective [2,3]. An estimated 10 million active TB cases were recorded in 2020, which consisted of 5.6 million men, 3.3 million women, and 1.1 million children [4]. More than 95% of TB deaths occurred in low- and middle-income countries (LMICs), with 8 LMICs accounting for two-thirds of the total cases, with India having the highest number of cases, followed by Indonesia, China, the Philippines, Pakistan, Nigeria, Bangladesh, and South Africa [1].

As the global population ages, LMICs will face an increasing brunt derived from noncommunicable diseases (NCDs). As defined by the World Health Organization (WHO), NCDs are chronic diseases lasting for a long duration and are a result of a combination of genetic, physiological, environmental, and behavioural factors [5]. NCDs, such as diabetes mellitus (DM), cardiovascular disease (CVD), chronic lung disease, cancer, and mental health illness (MHI) among others, are increasing in prevalence [6]. Each year, 41 million people die from NCDs, with 85% of these deaths taking place in LMICs [5]. In particular, CVDs account for 17.9 million deaths, followed by cancer at 9.3 million and DM at 1.5 million [5].

Global population health data posit that progress towards meeting TB control targets, including the expansion of TB intervention programmes, is hindered by the synergistic relationship between TB and NCDs [7,8]. This is strongly attributed to NCDs and their associated risk factors such as CVD, DM, and smoking and alcohol abuse being risk factors for TB, consequently impacting the burden of TB at the population level [7,9].

At the societal level, the TB and NCDs share numerous underlying social determinants, such as high levels of poverty, poor health literacy, social and cultural values, and lack of social protection [10–13]. Additionally, coexisting communicable and NCDs increases the risk or effect of each other [14]. However, the current healthcare systems of LMICs might not have sufficient resources to respond to this combined burden of diseases derived from communicable and NCDs [15]. At the same time, disease-specific interventions are not the most effective approach for tackling these afflictions [16,17].

A known example is DM, which significantly increases the risk of TB and vice versa. The tight interplay between disease conditions at the biological level complicates the management of TB and contributes to poor TB treatment outcomes [18]. Concomitantly, TB can increase the risk of or aggravate DM status [19]. Other known linkages between TB and NCD typologies include that of CVD and TB [20], chronic lung disease and TB [21], MHI and TB [22,23], and cancer and TB [24]. These mutual predispositions necessitate better integration of health services to place TB and NCDs under control in LMICs after accounting for specific disease burdens within each country context.

Therefore, there are multiple ways in which service integration can be manifested. Integration covers disease-specific (vertical) programmes and system-wide (horizontal) pillars and initiatives, overlaying intersecting disease services (e.g., DM and TB treatment services), across community and tertiary level interventions, and between health and nonhealth sectors. This review focuses on integrating service delivery for diseases that are usually delivered separately but often affect the same types of end users. We also acknowledge that service integration goes across and beyond the status quo of a single healthcare entity or stakeholder and have therefore not only consolidated information on the different models of service integration but also provide practical steps (incorporating facilitators and avoiding barriers) when implementing TB and NCD integrated programmes.

Despite the increasing burden of disease derived from TB and NCDs in LMICs and the need for TB and NCD integrated programmes, most systematic reviews on TB service integration investigate the integration of TB- and HIV-related services or the health outcomes of TB and NCD comorbidities, their syndemic interactions, and prevalence [25–34]. Therefore, there remains a dearth of information regarding the mechanism of integration of TB and NCDs related services in LMICs, which this systematic review aims to address. Hereafter, this systematic review will present integration models that bring together the different services for TB and NCDs; points of service delivery; process modifications; and change management strategies through a quantitative and qualitative lens.

## Methods

A protocol for this study has been registered on the PROSPERO international prospective register of systematic reviews (registration number: PROSPERO CRD42020202745). This study is reported as per the Preferred Reporting Items for Systematic Reviews and Meta-Analyses (PRISMA) guidelines (S1 and S2 Checklists) and illustrated in a flowchart below.

First, the findings from the literature review will be summarised based on its depth of integration, from least to most integrated, with each being delved into detail. A framework that illustrates the models of service integration will be developed from this step. Second, using our organised findings from the first step, WHO’s 6 operational “building blocks” of a health system, a well-established framework that identifies health system facilitators and constraints will be used to understand the facilitators and barriers for each integration model [35]. This step involves mapping various integration characteristics derived from the literature review to each dimension of the framework.

Drawing on the definitions proposed by Atun and colleagues [36], Dudley and Garner [16], and Legido-Quigley and colleagues [37], we operationally define programmatic integration as managerial or operational reorganising of health systems components to consolidate inputs, delivery, management, and organisation of particular service functions as a means to improve coverage, access, quality, acceptability, and (cost)-effectiveness [38]. For the purposes of this review, we consider the various tenets of integration that encompass the integration of service provision for both TB- and NCD-related services in terms of physical point-of-care delivery and types of services that include screening, testing or referral, and nonmedically related services like financial and social support.

### Study selection

#### Type of chronic conditions

To be considered for inclusion in the current review, the studies had to integrate TB with NCDs. The list of NCDs included are chronic CVDs (coronary heart disease, cerebrovascular disease, peripheral arterial disease, and hypertension), DM (type 1 and 2), cancers (breast cancer, cervical cancer, colorectal cancer, prostatic cancer, and lung cancer), chronic obstructive pulmonary disease (asthma, chronic bronchitis, and emphysema), and mental health conditions (depressive disorders, vascular dementia, and Alzheimer disease but exclusive of alcohol and substance misuse). We have focused on the integration of care of the established diseases and excluded the risk factors of the diseases for this study.

#### Inclusion criteria

Publications were included if they described or evaluated management or organisational change strategy made within an existing health system to increase integration between TB and NCD management at the service delivery level in LMICs. Services could be provided in health facilities or the community. Reports had to describe the experience of integration, not a theoretical account of how integration might be implemented. We included articles that studied populations of all age groups at the community, primary, secondary, or tertiary care level or venues that we have detailed in the tables below. We also included articles irrespective of drug sensitivities. The extracted articles included both qualitative and quantitative studies and their subcategories, including but not limited to retrospective and prospective cohort, cross-sectional, case–control, ethnographic, randomised control, and observational studies. We also reviewed studies conducted in languages other than English.

#### Exclusion criteria

To ensure a comprehensive descriptive review, we did not exclude studies based on their design or absence of outcome measures. Health promotion or disease prevention papers were excluded even if the activity was delivered in a TB or NCD treatment setting. We excluded opinion, editorial, and correspondence pieces on care integration without actual implementation of integrated programmes. Studies not conducted in the LMICs were also excluded.

#### Search strategy

The search strategy was developed to be consistent with methods used by other authors for systematic reviews of integration of health services. We searched databases such as the Cochrane Library, MEDLINE, Embase, PubMed, Bibliography of Asian Studies, and the Global Index Medicus from their inception to November 4, 2021. The search terms used for these databases are shown in Box 1.

**Box 1. Search strategy used for the Cochrane Library <1945 to November 4, 2021>, Ovid MEDLINE(R) <1946 to November 4, 2021>, Embase <1974 to November 4, 2021>PubMed<1950 to November 4, 2021>, Bibliography of Asian Studies <1946 to November 4, 2021>, and the Global Index Medicus <inception to November 4, 2021>**

1. (vertical or horizontal or integrat* or coordinat* or co-ordinat*).tw. or delivery of healthcare, integrated/ or primary healthcare/ or secondary healthcare / or tertiary healthcare/ or community healthcare.tw.
2. exp Tuberculosis/ or (tuberculo* or tb).tw.
3. (All introduced in a separate line) (chronic disease or (chronic* adj3 (disease* or disab* or ill* or condition* or health condition* or medical condition*)) or (noncommunicable disease* or noncommunicable disease* or NCD or NCDs)).tw. or *cerebrovascular disorders/ or (cerebrovascular disease* or cerebrovascular disorder* or brain ischaemia or cerebral infarction or carotid artery disease* or stroke).tw. or exp myocardial ischemia/ or (myocardial isch* or ischaemic heart disease or ischemic heart disease or angina or coronary disease* or coronary heart disease* or coronary artery disease* or myocardial infarction).tw. or exp heart failure/ or heart failure.tw. or hypertention.tw. or high blood pressure.tw. or *Diabetes Mellitus, Type 1/ or *Diabetes Mellitus, Type 2/ or exp lung diseases obstructive/ or (obstructive lung disease* or obstructive pulmonary disease* or asthma or bronchitis).tw. or exp emphysema/ or exp pulmonary emphysema/ or emphysema.tw. or exp neoplasms/ or (cancer* or oncolog* or neoplasm* or carcinom* or tumor* or malignan*).tw. or exp mental disorder/ or mental health.tw. or depression*.tw. or exp dementia/ or (dementia or alzheimer*).tw. or exp anxiety/ or anxiety*.tw. or exp epilepsy/ or epilep*.tw.
4. 1, 2, and 3.

We conducted hand searching and consulted expert reviewers to check the completeness of the electronic searches and included additional papers as appropriate. The World Bank List of Economies 2019 was utilised as reference of the LMICs for the study [39]. The list of the LMICs was not included in the search term, but articles resulting from the database search were reviewed for LMICs during the assessment of the full articles.

### Search and retrieval of studies

Two reviewers (PS and LT) independently went through the list of articles from the electronic database search results to identify articles relevant to this systematic review based on title or title and abstract. If either of the 2 reviewers considered a study potentially eligible, the full article was retrieved for further assessment. Further potential studies were also identified by screening the reference lists of included articles. Two reviewers assessed the full texts independently to evaluate whether they met the inclusion criteria for this review. Any disagreements concerning studies to be included were resolved by consensus or by discussion with a third reviewer (QD). There were no language restrictions.

### Data extraction

A data extraction format was developed in Microsoft Excel. Data were extracted from each study on the country, study setting, duration of integration programme, model of integration, entry into the health system, screening tools used, study methods, outcomes, and facilitators and barriers to programme implementation. Data extraction was performed independently by 2 reviewers (PS and LT) and compared. To further ensure accuracy, a third reviewer (CDF) not involved in searching and retrieving studies checked for disparities. Erroneous and inconsistent data sets reported in the studies were excluded. However, no discrepancies in data were identified.

### Quality assessment (risk of bias)

Included studies were independently assessed for quality and risk of bias by 3 reviewers (PS, QD, and CDF). The quality (risk bias) assessment tools used were selected and adapted from a review of tools performed by Ma and colleagues, which provides an overview of the relevant tools to evaluate methodological quality for different types of studies [40]. Therefore, Newcastle–Ottawa Quality Assessment Form for Cohort Studies was employed for retrospective and prospective cohort studies [41]. For case–control studies, Scottish Intercollegiate Guidelines Network (SIGN) critical appraisal checklists for case–control studies was used [42]. For cross-sectional studies, Joanna Briggs Institute (JBI) Critical Appraisal Checklist for Analytical Cross-Sectional Studies tool was deployed [43]. For mixed methods studies, the Mixed Methods Appraisal Tool was used [44]. The most relevant and salient features extracted from each study based on the aforementioned tools are reported in the tables below.

### Data analysis and synthesis of results

First, we presented the relevant quantitative and qualitative findings in the tables below. As such, we reported the quantitative indicators such as relative risks, odds ratios, prevalence ratios, and prevalence with their associated 95% confidence intervals and number needed to screen and number needed to test where appropriate. We also reported qualitative findings that included the perspectives and accounts of patients and providers regarding the TB and NCD integration programmes, which they were a part of.

Second, 4 reviewers (PS, LT, QD, and CDF) reviewed the data and organised the findings to develop a new framework of integrated services for TB and NCDs through relevant themes extracted from the reviewed studies. To that end, a previously published and validated framework for integrating TB and HIV service delivery by Legido-Quigley and colleagues was reconfigured and updated to address the salient features of integrative TB- and NCD-related services [37]. In the original framework by Legido-Quigley and colleagues, users enter the health system through TB services, where in the least integrated model, the users are referred to other sites for HIV testing and subsequent care. With closer integration, TB clinics can also test for HIV on-site but continue to refer to another service for HIV treatment. Where users enter through HIV services, in the least integrated model, the users are referred to a TB clinic for screening for active TB; in a more integrated model, TB screening is undertaken within the HIV clinic, but users are referred to other sites for TB treatment services. In the most integrated model, treatment for both HIV and TB is provided in one health facility. Hence, the different levels of integration and the types of services provided were concepts drawn from the original framework and incorporated into the newly synthesised TB and NCDs framework below.

Third, the 4 reviewers (PS, LT, QD, and CDF) reanalysed the data thematically to derive themes on the facilitators and barriers for TB and NCD service integration and subsequently mapped the themes into WHO’s 6 operational “building blocks” of a health system framework.

However, given the significant heterogeneity in study designs, participants, contexts, and outcomes reported in each study, we were unable to perform meta-analyses. Instead, a descriptive synthesis was performed.

## Results

### Overview of included studies

**Fig 1:**
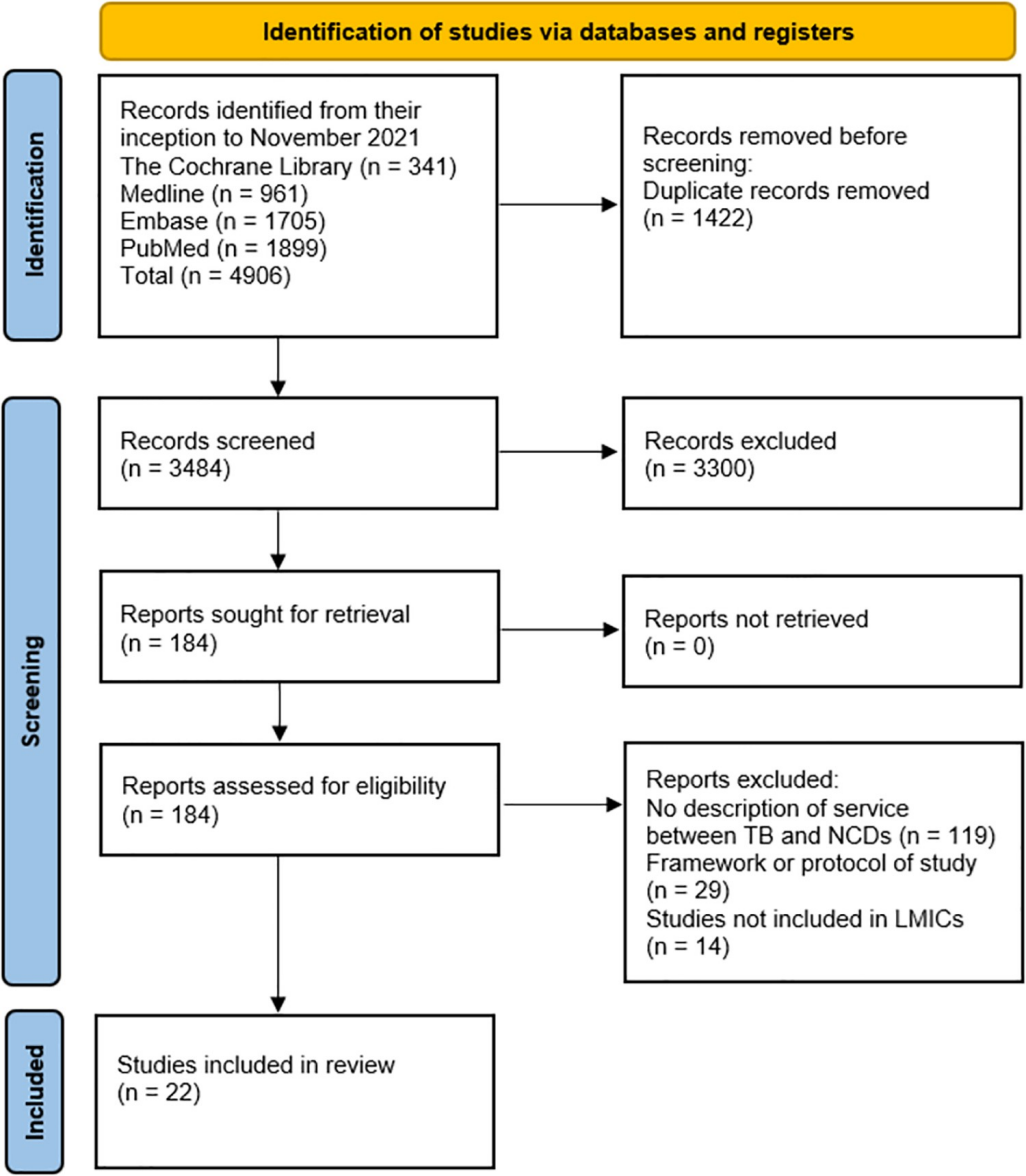
PRISMA 2020 flow diagram of the study selection process. LMIC, low- and middle-income country; NCD, noncommunicable disease; PRISMA, Preferred Reporting Items for Systematic Reviews and Meta-Analyses; TB, tuberculosis.

The screening process of this review adapted the PRISMA flowchart (Fig 1). Our database search yielded 4,906 articles. After the removal of duplicates, 3,484 articles were screened for titles and abstracts. A total of 184 full articles on integration of care of TB and NCDs were further assessed for eligibility, and 22 articles met the inclusion criteria for this review.

### Characteristics of included studies

Of the 22 articles included, 13 were cross-sectional studies, 3 were cohort studies, 1 was a case–control study, 1 prospective interventional study, and 4 were mixed methods studies. A brief summary of the key characteristics of each study is provided in Table 1 below. Seven studies had a low risk of bias, 11 studies had a moderate risk of bias, 3 studies had a high risk of bias, and 1 study did not have enough data for risk bias analysis (elaborated in Tables 2–6).

**Table 1:** Description of the studies included.

Author/year	Country	Duration of programme/intervention	Study design	Study setting	Entry to system via	Level of integration	Diseases included
Byrne AL, 2018	Peru	2014 to 2015	Case–control study	Central Lima districts of Cercado and La Victoria, Peru	TB	Level 1 test	TB Hypertension DM CKD
Dasa TT, 2019	Ethiopia	February to July 2017	Institution-based cross-sectional study	8 hospitals and 3 health centres in eastern Ethiopia	TB	Level 1 test	TB MHI
Masumoto S, 2014	Philippines	September to November 2012	Cross-sectional study	10 public health centres and 2 NGO clinics providing DOT in District I, Tondo, Manila	TB	Level 1 test	TB MHI
Peltzer K, 2018	South Africa	May to October 2011	Cross-sectional study	42 primary care clinics in 3 districts (Siyanda, Nelson Mandela Metro, and eThekwini)	TB	Level 1 test	TB Myocardial infarction Arthritis Asthma Chronic lung disease DM Hypertension Dyslipidemia Malignant neoplasms
Peltzer K, 2012	South Africa	April 2011 to mid-October 2011	Cross-sectional study	42 primary care clinics in 3 districts (Siyanda, Nelson Mandela Metro, and eThekwini)	TB	Level 1 test	TB MHI
Peltzer K, 2013	South Africa	Mid-April to mid-October 2011	Cross-sectional study	42 primary care clinics in 3 districts (Siyanda, Nelson Mandela Metro, and eThekwini)	TB	Level 1 test	TB MHI
Restrepo B, 2011	Mexico	March 2006 to September 2008	Cross-sectional prospective study	TB clinics in Hidalgo and Cameron County Health Departments in eastern Mexico	TB	Level 1 test	TB DM
Segafredo G, 2019	Angola	January 2015 to December 2016	Cross-sectional study	6 directly observed DOT centres in Luanda, Angola	TB	Level 1 test	TB DM Hypertension
Anand T, 2018	India	October 2016 to March 2017	A mixed methods methodology was taken: (1) cross-sectional study; and (2) a qualitative component using a descriptive study approach using interview data	2 DOT centres attached to 2 medical colleges in Delhi	TB	Level 2 test and refer	TB DM Hypertension
Contreras CC, 2017	Peru	September 2015 to May 2016	A descriptive cross-sectional study	9 public health facilities supported by TB Cero Clinic in Carabayllo	TB	Level 2 test and refer	TB DM MHI HIV
Ekeke N, 2020	Nigeria	February to October 2018	Hospital-based cross-sectional study was done under programme implementation	Diabetes clinics of selected health facilities with high DM patient load and readily accessible DOT centre	NCDs	Level 2 test and refer	TB DM
Qader G, 2019	Afghanistan	May to December 2017	Cross-sectional study	5 public and 1 private health facility in Kabul, Jalalabad, Kandahar, Herat, and Mazar-e-Sharif provinces	NCDs	Level 2 test and refer	TB MHI
Zhang X, 2015	China	January to December 2013	Feasibility study of clinical intervention (cross-sectional study)	Primary care facilities in Qufu, Wendeng, and Zouping counties of Shandong Province	NCDs	Level 2 test and refer	TB DM
Govindasamy D, 2013	South Africa	March 2010 to September 2011	Observational cohort study	A mobile unit provided screening for HIV, TB symptoms, DM, and hypertension	TB and NCDs	Level 2 test and refer	TB DM Hypertension HIV
Jerene D, 2017	Ethiopia	February to June 2015	Cross-sectional study	4 secondary public hospitals in Amhara and Oromia regions of Ethiopia	TB and NCDs	Level 2 test and refer	TB DM HIV
Araia ZZ, 2021	Eritrea	January 2016 to December 2019	Cross-sectional study	2 community hospitals and 8 health centres in Maekel region, Eritrea	TB and NCDs	Level 2 test and refer	TB DM
de Castro-Silva KM, 2019	Brazil	July 2015 to December 2016	Cross-sectional, descriptive, survey-based study	Municipal Health Centres (Centro Municipal de Saúde) of Duque de Caxias, Rio de Janeiro	TB	Level 3 colocated test and treatment	TB MHI
Gnanasan S, 2011	Malaysia	November 2009 to February 2010	Mixed methods study with a descriptive quantitative methods and qualitative interviews	A tertiary hospital in the northern region of Peninsular Malaysia	TB	Level 3 colocated test and treatment	TB DM
Lovero KL, 2019	South Africa	November 2016 to July 2017	Mixed methods exploratory design for programmatic evaluation	40 clinics (10 per district)	TB	Level 3 colocated test and treatment	TB MHI
Peltzer K, 2016	South Africa	**-**	Longitudinal observational study (cohort study)	40 primary healthcare facilities	TB	Level 3 colocated test and treatment	TB MHI
Walker IF, 2018	Nepal	March 2015 to May 2016	Feasibility study using mixed methods design	2 NTP MDR-TB treatment centres and 8 subcentres in Nepal	TB	Level 3 colocated test and treatment	TB MHI
Pasha A, 2021	Pakistan	February 2017 to June 2018	Prospective interventional study	1 private tertiary care hospital, 3 public sector tertiary care hospitals, and 2 private sector diagnostic and treatment centres in Karachi, Pakistan	TB	Level 3 colocated test and treatment	TB MHI

CKD, chronic kidney disease; DM, diabetes mellitus; DOT, direct observation therapy; HIV, human immunodeficiency virus; MDR-TB, multidrug-resistant tuberculosis; MHI, mental health illness; NCD, noncommunicable disease; NGO, nongovernmental organisation; NTP, National Tuberculosis Programme; TB, tuberculosis.

**Table 2:** Summary of level 1 integration, entry via TB.

Author/year/country	Sample characteristics	Outcome measured	Summary of the integration model	Screening tools used	Summary of study finding	Quality assessment (risk bias)
Byrne AL, 2018, Peru	177 participants with TB patients and 161 community controls aged between 10 and 70 years, of which 57.6% were male, and 42.4% were female	Prevalence of NCDs in TB and non-TB groups NNS for new NCDs	TB group who had successfully completed treatment for pulmonary TB and non-TB group were screened for DM, hypertension, and CKD. Screening entailed taking a medical history and performing ambulatory blood pressure measurement and urinalysis	A questionnaire on basic medical history and sociodemographic data Anthropometric measurements Clinical assessment with standardised measurement of resting BP was conducted in accordance with American Heart Association A multipanel dipstick test for urinalysis	Self-reported DM was almost 4-fold higher in the TB group than the non-TB group (aOR 3.66, 95% confidence interval 1.68 to 8.01), whereas no significant difference in the prevalence of CKD in both the groups. The NNS to find one new case of hypertension or proteinuria in the TB group was 24 and 5, respectively. The authors reported the feasibility for TB control programmes to incorporate routine screening and secondary prevention of common NCDs	Moderate risk of bias overall as: (1) The number of participants lost to follow-up and declined participation was much smaller compared to the total sample size of both case and control groups. (2) A representative sample of controls were recruited from the same community. (3) Potential recall bias among positive TB cases as they are more likely to recall a previous NCD diagnosis as compared to a participant without TB. (4) Possibility of misclassification bias due to the limitations of urinalysis testing for DM and CKD.
Restrepo B, 2011, Mexico	172 TB patients aged 20 years and above, with the mean age of 43 years for participants from NE Mexico	Prevalence of NCD (DM)	Existing and newly diagnosed TB patients were screened for DM with the objective of estimating the risk of TB attributable to DM in the population along the Mexican border	American Diabetes Association classification guidelines for epidemiological studies: hyperglycemia and/or self-reported DM Blood anticoagulated with EDTA and a handheld glucometer to screen for DM	Patients with DM were at higher risk of contracting TB than non-DM patients. Patients with DM were aware of having diabetes for approximately 8 years before their TB diagnosis and were suggested to be more likely to report comorbidities classically associated with DM than patients without DM. The relative risk of TB to DM in NE Mexico is RR 3.1, 95% confidence interval 2.3 to 4.2. The infrastructure for TB control could serve to improve the early detection of DM in TB patients, particularly in developing countries. The authors also highlighted that DM control worldwide could be significantly enhanced by the sharing of resources, experience, and infrastructure among resource-limited programmes for TB and DM control	High risk of bias overall as: (1) Potential overestimation of the prevalence of DM as TB can be caused by transient hyperglycemia. (2) Inherent biases in data sets might be amplified when the study merged data sets from different population sources. (3) TB infection was not confirmed by culture screening in a substantial number of participants, which might have led to an overreporting of actual TB cases.
Dasa TT, 2019, Ethiopia	403 TB patients undergoing anti-TB treatment for more than 1 month were recruited with an age range between 7 and 74 years, of which 59.3% were male, and 40.7% were female	Prevalence of NCD (depression)	TB patients under anti-TB treatments for more than 1 month were screened for MHI depression disorder	PHQ-9 to screen for depression	The prevalence of depression among TB patients was 51.9%, 95% confidence interval 42.7% to 62.2%. Newly diagnosed TB patients were also associated with depression with aOR 0.39. 95% confidence interval 0.21 to 0.74	Low risk of bias overall as: (1) Sample is representative of the population, and the nonresponse rate was 5%. (2) PHQ-9 tool had been translated into local languages and validated before deployment.
Masumoto S, 2014, Philippines	561 TB patients, of which 65.4% were male, and 34.6% were female, with the mean age of 41.87 years	Proportion with NCD (depression)	TB patients were screened for MHI depression at public health centres and NGO clinics providing DOT care	PHQ-9 to screen for depression The Duke-UNC FSSQ The MRC dyspnoea scale	The prevalence of depression was 16.8% and factors associated with depression were BMI < 18.5 kg/m^2^ (aOR 2.16, 95% confidence interval 1.25 to 3.73) and low perceived confidant social support (aOR 2.16, 95% confidence interval 1.08 to 4.31) among others. Depression among TB patients in poorer regions and with low education level is common, and screening for depression in primary care settings is an opportunity to identify patients needing support and treatment, especially for malnourished patients and those with poor social support	Low risk of bias overall as: (1) Patient information was collected from TB register and NTP treatment cards at the health facilities, reducing the likelihood of recall bias. (2) Questionnaire was translated into Tagalog from English using forwards–backwards translation method to ensure accuracy. A pretest was also conducted to confirm the feasibility and validity of the questionnaire. (3) Study was conducted using face-to-face interviews, which may result in misclassification of smoking status, alcohol consumption, and exposure to secondhand smoke due to social desirability bias.
Peltzer K, 2018, South Africa	4,207 TB patients between the age range of 18 to 93 years, of which 53.5% were male, and 46.5% were female	Proportion with common NCDs Proportion receiving NCD-related treatment	TB patients aged 18 to 93 years were screened for MHI depression and their risk factors at 42 primary healthcare facilities	10-item AUDIT General physical and mental health were assessed with the SF-12 K-10 was used to measure anxiety or depressive disorders PC-PTSD Screen was used to assess PTSD symptoms Participants were asked if they had NCDs and if they were on any NCD-related treatment. No particular tool was used for the screening of nonmental health-related conditions	The prevalence of comorbidity (with 1 NCD) was 26.9% and multimorbidity (with 2 or more NCDs) was 25.3%. The likelihood of multimorbidity was higher in those 45 years and above with aOR 2.48, 95% confidence interval 1.66 to 2.31, among men with aOR 2.34, 95% confidence interval 1.93 to 2.84, suffering from higher levels of poverty as compared to low levels of poverty with aOR 1.81, 95% confidence interval 1.36 to 2.41 but was lower in those with higher education of grade 12 and above compared to Grade 7 or less with aOR 0.4, 95% confidence interval 0.3 to 0.53. The authors suggested having primary healthcare delivery move away from single chronic condition focused management to a more integrated care approach that manages TB and NCDs holistically	Moderate risk of bias overall as: (1) Tools used for screening had a high Cronbach’s Alpha. (2) All analysis models were adjusted for confounders. (3) Study was limited to 3 selected districts and might not be representative of the entire South African population. (4) Study used a count of NCDs as a measure of multimorbidity, implying that each chronic condition has an equal impact on an individual, whereas in reality, disease severity, the specific combination of NCDs, and access to healthcare affect multimorbidity.
Peltzer K, 2012, South Africa	4,900 TB patients between the age range of 18 to 93 years, of which 54.5 % were male, and 45.5 % were female	Proportion with common MHIs Proportion having TB (self-reported) Proportion that adhered to TB medication regiment Proportion on antidepression medication	TB patients were screened for common MHIs, in particular psychological distress at 42 primary healthcare facilities	K-10 to measure psychological distress 10-item AUDIT Poverty screening tool was operationalised	Using a cutoff score of ≥28 and ≥16 on the K-10, 32.9% and 81% of TB patients had symptoms of distress, respectively. In the multivariable analysis, older age (aOR 1.52, 95% confidence interval 1.24 to 1.85), lower formal education (aOR 0.77, 95% confidence interval 0.65 to 0.91), poverty (aOR 1.90, 95% confidence interval 1.57 to 2.31), and not married, separated, divorced, or widowed (aOR 0.74. 95% confidence interval 0.62 to 0.87) were associated with psychological distress (K-10 ≥28), and older age (aOR 1.30, 95% confidence interval 1.00 to 1.69), lower formal education (aOR 0.55, 95% confidence interval 0.42 to 0.71), and poverty (aOR 2.02, 95% confidence 1.50 to 2.70) were associated with psychological distress (K-10 ≥16). The authors suggested improved training of providers in screening for psychological distress, appropriate referral to relevant health practitioners and providing comprehensive treatment for patients with TB and NCDs	Moderate risk of bias overall as: (1) Questionnaires were forwards and backwards translated into the major languages of study participants (Afrikaans, Tswana, Xhosa, and Zulu). (2) Tools used for screening had a high Cronbach’s Alpha. (3) Only 0.7% of the total people approached refused participation. (4) Kessler scales had significantly lower discriminating ability for depression and anxiety disorders among the Black African population than the combined non-Black African population group in South Africa due to differential item biased measurement.
Peltzer K, 2013, South Africa	4,900 TB patients between the age range of 18 to 93 years, of which 54.5 % were male, and 45.5 % were female	Proportion with common NCDs (MHI) Proportion of patients having TB (self-reported) Proportion that adhered to TB medication regiment Proportion on antidepression medication	TB patients were screened for MHI PTSD at 42 primary healthcare facilities	Primary Care PC-PTSD Screen to assess PTSD symptoms K-10 to measure psychological distress 10-item AUDIT	The study found that 29.6% of TB patients screened positive for PTSD symptoms. Positive screening for PTSD among TB patients was higher in people living with higher levels of poverty aOR 1.66, 95% confidence interval 1.5 to 2.19, people living in urban areas compared to rural areas with aOR 2.92, 95% confidence interval 1.96 to 4.3 and people with HIV and TB compared to those with no other chronic conditions with an aOR 1.28, 95% confidence interval 1.12 to 1.46. Therefore, authors suggested strengthening healthcare systems to improve mental healthcare delivery by focusing on existing programmes and activities, such as those that address the prevention and treatment of TB and HIV	Moderate risk of bias overall as: (1) Questionnaires were forwards and backwards translated into the major languages of study participants (Afrikaans, Tswana, Xhosa, and Zulu). (2) Tools used for screening had a high Cronbach’s Alpha. (3) Only 0.7% of the total people approached refused participation.
Segafredo G, 2019, Angola	7,205 participants above the age of 15 years whereby 49.9% were male, and 50.15 were female	Prevalence of TB Prevalence of DM and hypertension	Patients screened and diagnosed for TB and attending the DOT programme received further screening for DM and hypertension. Patients also received proper and exhaustive information on risk factors for DM, hypertension, and how to lead a healthy lifestyle by community health workers at DOT centres	TB diagnosis was made clinically and/or with sputum microscopy Fasting plasma glucose or RPG with a glucometer to screen for DM Capillary blood glucose was determined using blood glucose metres Uncontrolled hypertension was tested using an automatic digital blood pressure monitor	The crude prevalence of DM among patients with TB was 6%. The crude prevalence rate for DM was slightly higher in male (6.3%) than female (5.7%) TB patients. Of the participants diagnosed with hypertension, more women (23%) had uncontrolled blood pressure than men (16%). The authors declared, although without actual evaluation, the feasibility of the integrated programme for the detection of hypertension and DM while acknowledging the need to further strengthen policies and guidelines as advocated in the Bali Declaration	Moderate risk of bias overall as: (1) Data were only collected from urban DOT clinics and is not representative of the entire population. (2) Only TB patients referred to DOT clinic were invited for screening. Thus, TB patients not referred to health services were not represented. (3) Used glucometers to measure blood glucose, which was not the most appropriate way to diagnose DM. Additionally, blood glucose measurement was performed only once.

aOR, adjusted odds ratio; aPR, adjusted prevalence ratio; AUDIT, Alcohol Use Disorder Identification Test; BP, blood pressure; CKD, chronic kidney disease; cPR, crude prevalence ratio; DM, diabetes mellitus; DOT, direct observation therapy; FSSQ, Functional Social Support Questionnaire; HIV, human immunodeficiency virus; K-10, Kessler Psychological Distress Scale; MDR-TB, multidrug-resistant tuberculosis; MHI, mental health illness; MRC, Medical Research Council; NCD, noncommunicable disease; NGO, nongovernmental organisation; NNS, number needed to screen; NNT, number needed to test; NTP, National Tuberculosis Programme; OR, odds ratio; PC-PTSD, Primary Care Post-Traumatic Stress Disorder; PHQ-9, Patient Health Questionnaire-9; PR, prevalence ratio; PTSD, post-traumatic stress disorder; RPG, random plasma glucose; RR, relative risk; SF-12, social functioning; TB, tuberculosis.

**Table 3:** Summary of level 2 integration, entry via TB.

Author/year/country	Sample characteristics	Outcome measured	Summary of integration model	Screening tools used	Summary of study finding	Quality assessment (risk bias)
Contreras CC, 2017, Peru	192 TB patients, of which 62% were male, and 38% were female with a median age of 32 years	Prevalence of medical comorbidities Proportion of households that need socioeconomic support Proportion of patients successfully linked to care or support Proportion of patients completing follow-up care Proportion of home-based treatment	TB Cero is a collaboration between the NGO SES, the Ministry of Health of Peru and the Municipality of Carabayllo. All patients diagnosed with TB in any of the 9 public health facilities in Carabayllo were referred to TB Cero upon diagnosis. Socioeconomic evaluations of the patients were conducted. Patients were referred to appropriate care at public health facilities covered by the NGO and provided with assistance to meet basic needs. Using services within the same facility was preferred, but patients were also referred to other providers if needed	HbA1c test to screen for DM SRQ-18 was used to identify MHIs (depression, psychotic disorders, or alcoholism) PHQ-9 to screen for depression Assessment tool for evaluating poverty and need for socioeconomic support	In the study population, 43% had at least 1 medical comorbidity other than TB. These included 4% of patients with HIV, 6% with DM, and 32% deemed at risk for a MHI. Of patients who required follow-up for a comorbidity, 100% initiated ART, 71% attended endocrinology consultations, and 66% attended psychology consultations. Additionally, out of the 65% of patients who completed the socioeconomic evaluation, 46% reported already receiving food baskets from the municipality, and 63% were given additional support, most commonly food vouchers and assistance in accessing healthcare. Therefore, as most TB patients were also suffering from other NCDs, the authors suggested the provision of nonmedically relevant services to patients due to the interlinkages between socioeconomic status and comorbidities.	Moderate risk of bias overall as: (1) Switch in the mental health tools used to evaluate mental health status from SRQ-18 to PHQ-9 in 2016 after the study had commenced. This might affect the accuracy of reporting. (2) Data request system was used to extract patient data, reducing the risk of recall bias by participants.
Anand T, 2018, India	Quantitative segment of the study: 403 participants with TB aged 20 years and above, of which 57% of were male, and 43% were female Qualitative segment of the study: 20 one-to-one interviews were conducted (16 with patients and 4 with health providers).	Prevalence of new cases of NCDs Proportion of patients referred out NNS for new cases	DOT centres were tasked with NCD-related screening for DM and hypertension. Patients who met the criteria for onwards care were referred internally to the NCD clinics within the same medical college hospital for follow-up treatment. Furthermore, patients who scored moderately on the screening tests were given information about prevention and control of NCDs without onwards referral for NCD services	WHO STEPS instrument used to collect NCD risk factor data Anthropometric measurements Blood (venous) fasting sugar levels of each patient were obtained from the existing patient medical records to screen for DM	The NNS for DM was 63, while 20 TB patients needed to be screened to get one new case of hypertension for stage 1 of screening using WHO STEPS instrument. The odds of developing NCDs in males compared to females is 1.9, 95% confidence interval 1.1 to 3.3, age 50 and above compared to age 20 to 34 is 7.0, 95% confidence interval 3.2 to 19. The study also reported qualitatively on the positive and negative experiences of patients and facilitators and challenges of healthcare providers.	Low risk for bias overall as: (1) Validated tools were used for data collectio,n and confounders were adjusted for during quantitative analysis. (2) Qualitative portion of the study made use of maximum variation sampling. (3) Demographic details of participants for interviews not provided.

aOR, adjusted odds ratio; aPR, adjusted prevalence ratio; ART, antiretroviral therapy; CKD, chronic kidney disease; cPR, crude prevalence ratio; DM, diabetes mellitus; DOT, direct observation therapy; HbA1c, hemoglobin; HIV, human immunodeficiency virus; MDR-TB, multidrug-resistant tuberculosis; MHI, mental health illness; NCD, noncommunicable disease; NGO, nongovernmental organisation; NNS, number needed to screen; NNT, number needed to test; OR, odds ratio; PHQ-9, Patient Health Questionnaire-9; PR, prevalence ratio; PTSD, post-traumatic stress disorder; RR, relative risk; SES, Socios En Salud; SRQ-18, Self-Reporting Questionnaire 18; STEPS, STEPwise Approach to NCD Risk Factor Surveillance; TB, tuberculosis.

**Table 4:** Summary of level 2 integration, entry via NCDs.

Author/year/country	Sample characteristics	Outcome measured	Summary of integration model	Screening tools CD	Summary of study finding	Quality assessment (risk bias)
Zhang X, 2015, China	93,094 elderly residents, of which 50% were male, and 50% were female, with an average age of 71.5 years	Prevalence of TB Prevalence of TB symptoms Proportion of patients following up for treatment after referral	Screening was conducted at the village level primary care facilities by a service team from the township hospital for routine NCD screening and general physical health screening. The examination for one village is usually completed within 1 week. Those who missed the opportunity for screening could go to the township hospital at their convenience to complete the examination. During the examination, each service team assigned one nurse to register all individuals at high risk of TB. A trained team member conducted a survey to record the participants’ symptoms, DM status, and close contact history. All identified individuals at high risk for TB were given a free CXR at the township hospital and those with CXR suggestive of TB were classified as TB suspects. All suspects were referred to the county TB dispensary for final diagnosis and follow-up consultation	Health examination CXR for diagnosis of TB	Out of the total population that went for screening, 9.7% were identified as high TB risk. Out of the population identified as high TB risk, 93.1% went for follow-up to get their CXR, and 6.9% had defaulted their follow-up. Out of the population identified as high TB risk, 0.9% tested positive for TB. The authors of the study concluded that integrating TB screening into routine health services in township hospitals in China will likely lead to optimal yield with regard to identifying positive TB cases with minimal incremental costs involved.	Low risk of bias overall as: (1) Findings might not be representative of the elderly residing in urban settings as the study was conducted in rural villages. (2) All diagnosis was made based on national TB guidelines based on clinical, microbiological, and radiological evidence.
Qader G, 2019, Afghanistan	8,073 participants whereby 29.6% were male, and 70.4% were female, with the mean age of 33.6 years and a median of 30 years	Proportion of patients diagnosed with TB NNS, NNT for new TB cases	The study included all patients diagnosed with MHIs and treated as either outpatients or inpatients and who were receiving follow-up care in the health facilities. Patients were screened using WHO criteria for TB by nurses when first presented at clinics. These patients were classified as having presumptive TB, and their sputum were sent for further testing at the laboratories. Those with other symptoms or who were found to be negative after GeneXpert testing were referred to a hospital physician trained TB management for further examination and diagnosis. All patients diagnosed with TB were referred and enrolled at a nearby DOT centre	Participants were interviewed for signs and symptoms of TB GeneXpert or clinical diagnosis of TB by a physician	The NNS to detect a single case of TB was 29.3. The NNTs were 6.1 and 19.3 for all forms of TB and bacteriologically confirmed TB, respectively. The overall prevalence of TB among mentally ill patients was 3.6%. Authors suggested that TB care and prevention services be integrated into mental health centres.	Moderate risk of bias overall as: (1) 6.6% of patient data on MHIs were missing and not included in the analysis. (2) Data collectors were trained in information extraction for MHI diagnosis from health cards and patient registers to increase the accuracy of data collected.
Ekeke N, 2020, Nigeria	3,457 patients were screened whereby 65.9% were male, and 34.1% were female, with a mean age of 59.9 years	Prevalence of TB NNS for new cases Proportion referred for TB treatment	DM clinics conducted symptomatic TB screening for all eligible DM patients using appropriate tools (checklist), identified presumptive TB cases among those screened for TB, collected 2 sputum specimens from identified presumptive TB cases, sent sputum specimens for GeneXpert diagnosis (second sputum specimen was equally processed if the first was negative), and subsequently referred all diagnosed TB cases to TB clinic for treatment	Xpert MTB/Rif assay for TB diagnosis Symptom-based standardised checklist for TB	Overall prevalence of TB was 0.8% in the population with DM and the NNS to make diagnosis of a TB case was 315 DM patients. The authors mentioned that the number of positive cases identified following screening, yield of TB cases and the NNS to make diagnosis of a TB showed prospect for similar integrated programmes.	Not enough data to assess the risk of bias.

aOR, adjusted odds ratio; aPR, adjusted prevalence ratio; CKD, chronic kidney disease; cPR, crude prevalence ratio; CXR, chest X-ray; DM, diabetes mellitus; DOT, direct observation therapy; HIV, human immunodeficiency virus; MDR-TB, multidrug-resistant tuberculosis; MHI, mental health illness; NCD, noncommunicable disease; NNS, number needed to screen; NNT, number needed to test; OR, odds ratio; PR, prevalence ratio; PTSD, post-traumatic stress disorder; RR, relative risk; TB, tuberculosis.

**Table 5:** Summary of level 2 integration, entry via TB and NCDs.

Author/year/country	Sample characteristics	Outcome measured	Summary of integration model	Screening tools	Summary of study finding	Quality assessment (risk bias)
Jerene D, 2017, Ethiopia	A total of 3,439 participants were recruited. DM patients with TB had a median age of 44 with interquartile range of (28 to 55), while TB patients with DM comorbidity and HIV coinfection had a median age of 30 with interquartile range of (22 to 45).	Proportion of patients with NCD (DM), TB, or HIV among those screened	At each of the 4 secondary hospitals selected for the study, a focal person was provided (who was responsible for site-level coordination) and 3 clinicians working in TB, ART, and DM clinics were involved in this programme. Clinicians in ART clinics screened patients both for TB and DM. Those in TB clinics screened patients for HIV and TB, and patients attending DM clinics were screened for TB. Referrals to available standard care services were also provided to positively screened patients for all diseases.	Risk scoring criteria to identify patients at risk of DM that was designed by the team for DM risk screening Fasting and RPG tests to confirm the diagnosis of DM Sputum microscopy was the preferred method of diagnosis for patients with productive cough Chest radiography was available for patients upon the clinician’s recommendation	In the study population, 0.7% of the patients with DM were detected for TB and 32.4% of patients with TB were detected for DM. The authors reported that tridirectional screening was feasible for detecting and managing previously undiagnosed TB, DM, and HIV. However, the authors also acknowledged that more work was needed to better understand the interaction between HIV and DM.	Moderate risk of bias overall as: (1) Risk scoring tool used to identify patients at risk of DM, adapted from a previous tool that was only validated in non-African populations. (2) Site focal person collected all completed checklists at the end of each work week and checked for completeness to ensure accuracy of the data sets. (3) Diagnosis of TB was mainly made through symptom screening followed by sputum microscopy. Since most DM patients with TB are asymptomatic, the study might underestimate the TB rate among patients with DM.
Govindasamy D, 2013, South Africa	9,806 participants whereby 41.5% were male, and 58.5% were female, with median age of 30 years with an interquartile range of 23 to 41.	Prevalence of HIV Prevalence of TB Prevalence of NCDs (DM and hypertension) Proportion of patients successfully linked to onwards care and commencing referred care	A mobile testing unit was used to provide health services for this mobile screening programme whereby patients waited to be screened in the mobile unit while an educator conducted health talks (i.e., on STIs, prevention of mother-to-child transmission, TB, DM, and hypertension). Following which, all clients then underwent client-initiated HIV testing, followed by TB symptom and NCD screening. Patients with TB and NCD comorbidities were followed up by telephone and referred to onwards care for NCD and TB management within 1 month of screening. Patients received a referral letter written by the nurse to facilitate linkage to a healthcare facility.	South African TB guidelines were used to identify persons suspected of TB Handheld glucometers used to screen for DM Electronic sphygmomanometers used to screen for hypertension An approved rapid screening test followed by approved confirmatory tests were used to screen for HIV	The disease-specific prevalence was 5.5% for newly diagnosed HIV-infected patients, 10.1% for TB suspects, 0.8% for newly diagnosed DM patients, and 58.1% for newly diagnosed hypertensive patients. Linkage to care for TB suspects, DM patients and hypertensive patients was 56.7%, 74.1%, and 50.0% respectively. However, the initiation of care for TB was only 9.7%, DM care was 60.9%, and hypertension care was 65.5%. The authors argued that the linkage to onwards care using a mobile testing unit was similar to facility-based screenings, showing that implementing mobile testing units for the provision of integrated screening and referral services is a viable model of care in LMIC settings.	Moderate risk of bias: (1) Individuals who were deceased, untraceable and those who attempted but failed to link to care, never linked to care or linked to care but not within the predefined period were categorised as not having linked to care, which led to underreporting of actual numbers that accessed linked care. (2) Data on the telephonic follow-ups conducted by the mobile unit counsellors 1 week postdiagnosis was of suboptimal quality, and thus the association of successful contact on linkage to care could not be assessed accurately. (3) Some contact details were lost by the staff of the mobile testing unit, resulting in not all eligible participants being reached. (4) Trained and experienced counsellors conducted follow-up sessions (telephone calls and/or home visits) to ascertain outcomes in the clients’ preferred language to minimise respondent bias.
Araia ZZ, 2021, Eritrea	1,134 TB patients whereby 53.6% were male, and 46.4% were female, having median age of 43 years with interquartile range of 30 to 60.	Prevalence of NCD (DM) Prevalence of TB	All TB patients diagnosed were screened for DM with fasting blood glucose test that took place at community hospitals and health centres. Patients suspected with DM were referred to DM clinic for confirmation of DM diagnosis, follow-ups, and appropriate management. Additionally, the DM clinic screened for potential TB cases and the positive cases were linked to TB services and treated according to standard TB management protocols.	Pretested structured data abstraction tool was used to collect TB treatment status and anthropometric measurements. Overnight fasting blood glucose test used to screen for DM AFB Microscopy and Xpert MTB/RIF used for diagnosis of TB	The prevalence of DM among TB patients was 9.9% and 54.5% of the patients with TB and DM were known DM cases and the rest were diagnosed during screening. In addition, 10.4% of TB cases were found to be in the prediabetes stage. Older patients aged 45 to 54 and ≥55 were associated with 4.9 and 7-fold odds (aOR 4.9, 95% confidence interval 1.4 to 16.9, *p* = 0.013) and (aOR 7.0, 95% confidence interval 2.1 to 23.0, *p* = 0.001) of having DM, respectively.	Moderate risk of bias overall as: (1) Potential overestimation of prevalence of TB as patients that were diagnosed without laboratory confirmation and physicians’ final decision on diagnosis were included in the study. (2) Temporary hyperglycemia in TB patients could occur as a result of a stress reaction to TB infection and hyperglycemic effect of some anti-TB drugs. But this hyperglycemic condition could partly or completely return to a normal glucose level after TB treatment completion. Hence, the DM and prediabetes prevalence in this study could have been overestimated as the new DM and prediabetes cases were not screened for DM after TB treatment completion.

AFB, acid-fast bacilli; aOR, adjusted odds ratio; aPR, adjusted prevalence ratio; ART, antiretroviral therapy; CKD, chronic kidney disease; cPR, crude prevalence ratio; DM, diabetes mellitus; DOT, direct observation therapy; HIV, human immunodeficiency virus; LMIC, low- and middle-income country; MDR-TB, multidrug-resistant tuberculosis; MHI, mental health illness; NCD, noncommunicable disease; NNS, number needed to screen; NNT, number needed to test; OR, odds ratio; PTSD, post-traumatic stress disorder; PR, prevalence ratio; RPG, random plasma glucose; RR, relative risk; STI, sexually transmitted infection; TB, tuberculosis.

**Table 6:** Summary of level 3 integration, entry via TB.

Author/year/country	Sample characteristics	Outcome measured	Summary of integration model	Screening tools used	Summary of study finding	Quality assessment (risk bias)
De Castro-Silva KM, 2019, Brazil	260 patients with prepulmonary TB whereby 36.1% were male, and 63.9% were female with mean age of 34.1 years.	Prevalence of TB Prevalence of NCD (depression)	All patients were screened for TB and offered treatment at the same facility (municipal health centre) if needed. After screening for depression, TB patients were referred to outpatient mental health services within the same municipal health centre.	PHQ-9 to screen for depression PHQ-9 scores ≥ 10 were subsequently assessed with the depression module of the MINI-Plus to confirm the diagnosis of depression Sociodemographic and clinical questionnaire used to collect data on sex, age, ethnicity, educational attainment, family income, signs, and symptoms of TB, smoking, alcohol and drug abuse, HIV infection, and other comorbid conditions Sputum smear for AFB and/or a positive rapid molecular test for *Mycobacterium tuberculosis* (Xpert MTB/RIF), chest radiograph were used to diagnose active TB cases.	TB was confirmed in 37.7% of the study population. A total of 60.2% of TB patients were screened positive for depression and 62.1% of non-TB patients screened positive for depression. Out of all patients that screened positive for depression, 54.6% were suffering from major depressive episode. The authors acknowledge that need for more studies to better understand the magnitude of comorbid TB and depression as well as to elucidate the sensitivity and specificity of the PHQ-9 among individuals affected by TB.	High risk of bias overall as: (1) PHQ-9 tool, which is usually self-administered, was administered by health providers instead due to poor literacy among study population which might have led to social desirability bias. (2) A high dropout rate of 24%, whereby patients did not return for sputum culture, making it impossible to diagnose TB accurately. Therefore, the prevalence of TB might have been underreported. (3) Authors acknowledged that other confounders such as BMI were not needed in the analysis and did not explicitly reveal which confounders were not included.
Gnanasan S, 2011, Malaysia	35 out of 53 patients identified to have both TB and NCD participated in the study with age range of 29 to 73 years, whereby 62.9% were male and 37.1% were female.	Number of medication nonadherence Number of uncontrolled DM Number of adverse drug reactions and individual patient’s medication-related problems	TB patients with DM were identified from patients’ medical records. The integrated model was meant to follow the 7 practice steps suggested by Hepler and Strand. Following these steps, the pharmacist established a relationship with the patient and monitored medication adherence and follow-up care for TB and DM management. Patients were educated and counselled on the management of TB and DM during their first visit. Some of the medication-related concerns were addressed verbally, and some were communicated to their physicians. All care processes took place in the same tertiary hospital.	Screening tools used not available as patients were diagnosed prior to enrolment. The model includes integration of TB and DM medication intervention tailored by clinical pharmacists to suit the individual patient’s needs.	The qualitative component of the study reported that pharmacists played an important role in integrating care for TB and DM by providing individualised pharmaceutical care management to address patients’ nondherence to medications, uncontrolled DM, and adverse drug reactions. However, the authors suggested further studies to evaluate the effectives of the programmes to impact overall health outcomes.	Low risk of bias overall as: (1) Pharmacist and researcher had weekly meetings to discuss barriers patients face in receiving care to consolidate their findings thus ensuring data collection accuracy and lower levels of recall bias.
Walker IF, 2018, Nepal	135 patients within the age range of 23 to 43 years, of which 76% were male and 2% were female.	Prevalence of NCDs (depression and anxiety) Proportion of patients receiving treatment (counselling and group sessions)	TB patients present at DOT clinic were provided with educational materials on MDR-TB and screened for depression and anxiety. HAP, which was an individual counselling initiative based on BA psychological therapy used for treating depression within the primary care setting using lay counsellors was provided to patients who met 2-stage screening criteria. Patients were also referred to physicians if needed. Patients were provided the option to participate in a support group if they wished. The support group aimed to increase patients’ understanding of MDR-TB and its treatment, reduces their negative emotions, and improves social support.	25-item Johns Hopkins Symptom Checklist to screen for depression and anxiety MSPSS to screen for low social support	In the study population, 9% received HAP counselling, 85% received information materials, 59% received an education session, and 36% received at least 1 group session. Eight group sessions were conducted in total. The qualitative component of the study reported that the intervention packages were acceptable to the patients, including the screening, information provided, support group and counselling services. The programme was also able to successfully train individuals with no prior experience of psychological counselling to deliver the HAP services.	Low risk of bias overall as: (1) 25-item Johns Hopkins Symptom Checklist was selected as it had previously been translated and culturally adapted for use Nepal for the identification of depression and anxiety among internally displaced people, has been shown to have moderate validity. (2) MSPSS tool was translated from English to Nepali by the research team and verified by back-translation. (3) Untested interrater reliability between the 2 counsellors that performed the screening at each centre might lead to discrepancies in screening.
Lovero KL, 2019, South Africa	85 participants, of which 59 nurses and 17 MHPs completed questionnaires in the study, and 9 DPMs were interviewed in the study.	Proportion of providers using a validated tool for screening Proportion of patients screened at every visit Proportion of patients screened every 3 months Proportion of patient diagnosed with MHI by MHPs Proportion of patients treated by MHPs Proportion of patients referred to onwards care	For patients who screen positive for MHIs, DPMs described a stepped-care approach in which MHPs diagnosed patients followed by treatment or referral to specialised care. But if the condition was not severe, MHPs could also treat them in the same primary care facility. MHPs are physicians, nurses, occupational therapists, psychologists, and social workers trained to provide prescribed mental healthcare, treatment, and rehabilitation services in primary care clinics. They could either be employed at the clinic full-time or on a visiting basis. After the patient’s condition is stable, a down-referral back to the original primary care clinic was performed for patients to be managed at the primary care level and maintain continuity of care with the same primary care provider. Patients with TB who visited the primary care clinics which provided the DOT services will be screened using PHQ-9. No referral to TB services for treatment was stated in the article. Evaluation of care integration was reported to have been conducted.	PC101—a South African clinical management tool that includes information on the screening, diagnosis, treatment, and referral of patients with MHIs Semistructured interviews conducted with DPMs and MHPs Survey to evaluate the integration of mental healthcare into primary care services	Only 73% of nurses reported conducting universal screening and 44% reported using a specific screening tool. Additionally, only 41% of MHPs indicated that they diagnose MHIs, and 82% offered any treatment for MHIs. The authors also concluded qualitatively that the integration of mental health services into existing health programmes was feasible if resources were available for the training and sustaining of the integrated programme. Furthermore, harmonising the agenda across various health initiatives within the country, increasing mental health awareness and ensuring downwards care referral to primary care providers for stable patients are cornerstones of the programme that could be improved.	High risk of bias overall as: (1) Not every clinics had an MHP available for participation in the study. Thus, findings might not be generalisable. (2) Qualitative coding process reported a high level of agreement between coders. (3) During the data collection phase, a very high-profile event involving the death of at least 94 mental health patients occurred. This could have caused an abnormal increase in mental health awareness, and participant responses might not have been representative of past procedures in patient management. (4) The purposive selection of clinics may have introduced bias. The study team and participating districts identified rural and urban clinics subjectively rather than using a specific metric to define these regions. (5) MHPs come from various professional backgrounds (e.g., psychiatrists, social workers, nurses, etc.), which might influence their level of comfort in managing mental illness and, in turn, the activities they perform in the management of mental illnesses.
Peltzer K, 2016, South Africa	710 TB patients, of which 71.9% were male and 28.1% were female, with 71.3% of the study population within the age range of 18 to 44 years.	Proportion with NCDs (depression and PTSD) Proportion receiving treatment for depression	TB patients receiving treatment at the primary care clinic were screened for common MHIs. Treatment for MHI was provided (details not mentioned in the study). Screening for mental health was then repeated at every 6-month interval.	General physical and mental health were assessed with the SF-12 K-10 to measure anxiety or depressive disorders PC-PTSD Screen to assess PTSD symptoms 10-item AUDIT Participants were asked if they had other NCDs and if they were on any related treatments. No particular tool was used for the screening of nonmental health-related conditions.	At baseline, 34.1% of the study population had severe psychological distress with a higher K-10 cutoff score (≥28), 81.1% had moderate psychological distress with a lower K-10 cutoff score (≥16), and 29.4% had PTSD symptoms (2 or more). At the 6-month follow-up, severe psychological distress with a higher K-10 cutoff score (≥28) was significantly reduced by 12.3%, moderate psychological distress with a lower K-10 cutoff score (≥16) was reduced by 24.9%, and PTSD was reduced by 20.0%. The authors commented that mental health services and social welfare services should be integrated into TB treatment programmes over a period of time to observe significant results.	Moderate risk of bias overall as: (1) Cronbach’s alpha for the K-10 screening tool was 0.92 at baseline and 0.87 at follow-up. (2) Cronbach for the PC-PTSD screening tool was 0.89 at baseline and 0.92 at follow-up. (3) More than twice the number of male than female participants; thus, the difference in screening and treatment outcomes between genders might not be pronounced. (4) Most study variables were assessed by interview, and socially desirable responses might have been provided. (5) Findings were limited to TB patients with alcohol problems on anti-TB treatment in public primary healthcare facilities.
Pasha A, 2021, Pakistan	3,500 TB patients whereby 51.4% were male and 48.6% were female, with the mean age of 34.8 years.	Proportion of patients with successful TB treatment Proportion of patients screened for MHIs (depression and anxiety) Proportion of patients that underwent mental health counselling	DS-TB patients were screened and treated for at public tertiary hospitals, private tertiary hospitals, and private diagnostic and treatment centres. IPUs offering mental health services were embedded into these TB treatment facilities. The mental health services were aligned with the TB treatment appointments.	AKUADS; a 25-item indigenously developed was used to assess anxiety and depression A sputum smear or culture test was used to test for active TB before and after treatment completion	Symptomatic patients who completed at least 4 counselling sessions had higher rates of TB treatment completion than those who did not (92.9% versus 75.1%; *p* < 0.0001). The authors argued that mental health interventions integrated within TB programmes can help reduce symptoms of depression and anxiety and improve TB treatment completion by having IPUs offer mental health services at TB treatment facilities.	Low risk of bias overall as: (1) AKUADS screening tool used, which was indigenously developed, was deployed in the local Urdu language and was validated in the local population. (2) High level of data consolidation allowed for 96.6% of patient TB treatment outcome measurements to be analysed.

AFB, acid-fast bacilli; AKUADS, Aga Khan University Anxiety and Depression Scale; aOR, adjusted odds ratio; aPR, adjusted prevalence ratio; AUDIT, Alcohol Use Disorder Identification Test; BA, behavioural activation; CKD, chronic kidney disease; cPR, crude prevalence ratio; DM, diabetes mellitus; DOT, direct observation therapy; DPM, district-level programme manager; DS-TB, drug-susceptible tuberculosis; HAP, Healthy Activity Program; HIV, human immunodeficiency virus; IPU, integrated practice unit; K-10, Kessler Psychological Distress Scale; MDR-TB, multidrug-resistant tuberculosis; MHI, mental health illness; MHP, mental health practitioner; MINI-Plus, Mini International Neuropsychiatric Interview; MSPSS, Multidimensional Scale of Perceived Social Support; NCD, noncommunicable disease; NNS, number needed to screen; NNT, number needed to test; OR, odds ratio; PC101, Primary Care 101 Guidelines; PC-PTSD, Primary Care Post-Traumatic Stress Disorder; PHQ-9, Patient Health Questionnaire-9; PR, prevalence ratio; PTSD, post-traumatic stress disorder; RR, relative risk; SF-12, social functioning; TB, tuberculosis.

**Fig 2:**
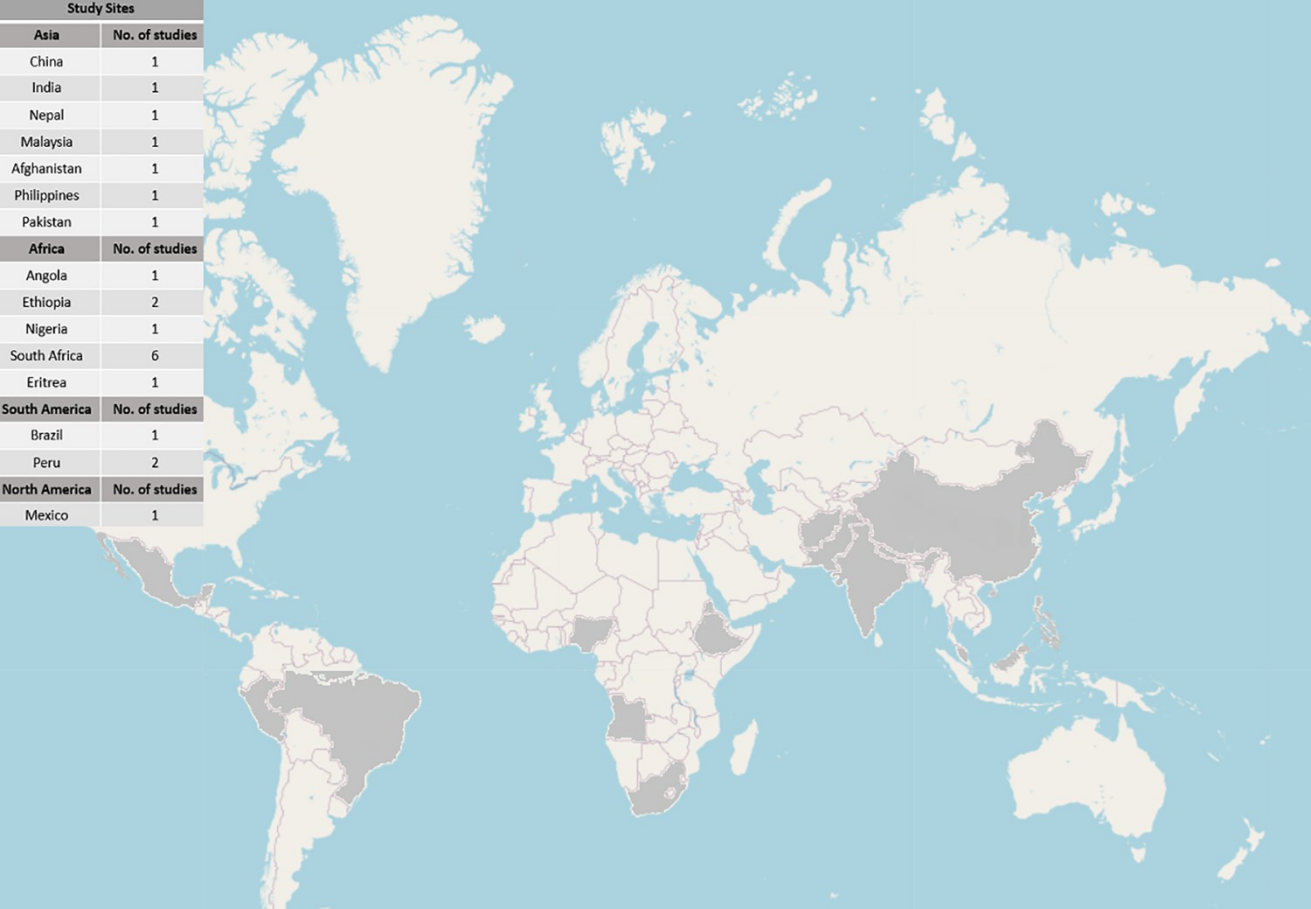
Map of study sites. Map shows the countries and the respective number of studies conducted in each country. The base layer of the map is taken from http://www.openstreetmap.org.

Seven studies were conducted in Asia, 11 in Africa, and 4 in the Americas (Fig 2). Studies conducted in Africa and Asia focused more on the integration of mental health care in TB patients, whereas studies from South America explored the integration of services for NCDs such as DM and hypertension in TB patients.

### Synthesised model of integration based on literature review

**Fig 3:**
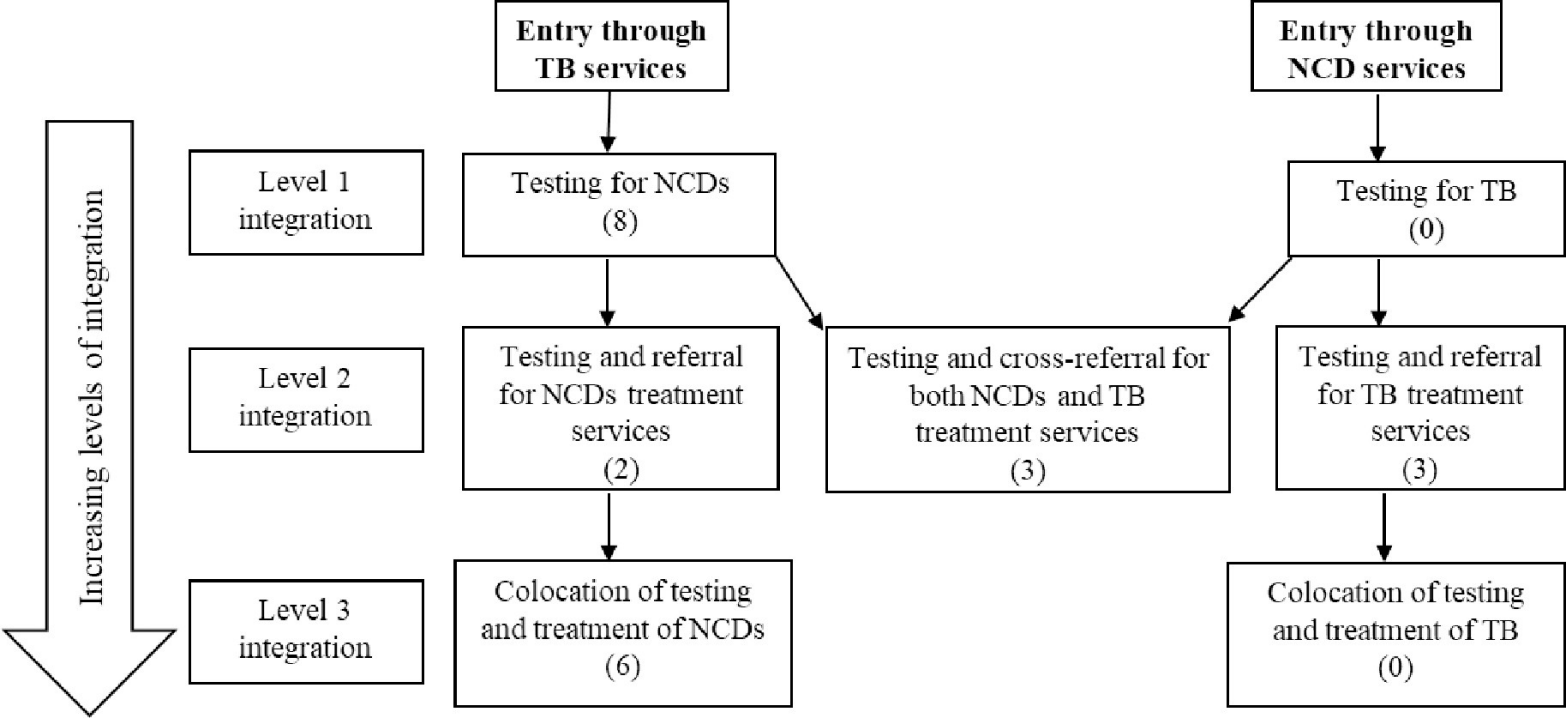
Framework for integration of TB and NCDs services. Illustrated summary of different models of integration using either TB or NCD as points of entry into the health system. Numbers in brackets represent the number of studies reporting the respective model of integration. NCD, noncommunicable disease; TB, tuberculosis.

A new framework of integrated care for TB and NCDs was developed where the depth of integration is stratified according to distinct levels 1, 2, and 3 in order to distinguish the integration of an additional service from the ones that are preexisting and/or stand-alone. The higher the numerical value, the deeper the level of service integration. Models of integration ranged from the type of services provided and point of entry into the healthcare system via TB or NCDs or both. We identified 7 models of integrated care and illustrated them in the framework below (Fig 3).

At level 1, the least integrated model, (i) patients entering the system via TB service are offered the additional service for screening for NCDs, and likewise (ii) the patients entering via NCDs service are screened for TB.

At level 2, moderate level of integration, (iii) patients entering from TB services are screened for NCDs and patients with potential or positive diagnosis are referred for care at appropriate healthcare facilities, and likewise (iv) patients entering the system from NCD services are screened and referred for TB treatment. Another model is also identified at this level, where (v) patients enter the system via both TB and NCDs services and are referred for the necessary additional TB- or NCD-related care after bidirectional screening is performed.

At level 3, the highest level of integration, (vi) patients entering the system via TB services are screened for NCDs, and (vii) patients entering the system via NCDs services are screened for TB and both models provide treatment or care management for both TB and NCDs at the same location (colocation).

The number of articles found at each level of integration is expressed in brackets, and entry points differentiated based on underlying known condition, be it TB or NCDs.

### Level 1 integration

#### Entry via TB service, testing for NCDs

This model was described in 8 studies whereby patients who entered the system by TB services were screened for 1 or more NCDs to establish the presence of comorbidity between TB and NCDs. In all 8 studies, existing and/or new TB patients were screened for NCDs at the location where patients received care for TB. A total of 5 studies screened for single NCDs, of which 4 screened for MHIs and 1 for DM. Three other studies screened TB patients for multiple NCDs such as DM, hypertension, and chronic kidney disease (CKD).

This integration model was reported in a case–control study by Byrne and colleagues in Peru, which explored the feasibility of screening for NCDs in patients receiving treatment for TB. In this study, the programme offered point-of-care screening for NCDs such as DM, hypertension, and CKD to 177 TB patients and 161 controls through the provision of detailed medical history taking, obtaining anthropometric measurements, clinical assessment with standardised measurements of resting blood pressure, and urinalysis. The authors reported the possibility of missing the diagnosis of CKD as the urinalysis procedures used offered only a moderate specificity of 53% as a shortfall in the study [45].

Similar issues of screening accuracy were raised in a cross-sectional study by Restrepo and colleagues in Mexico. This study aimed to estimate the risk of TB attributable to DM and identify opportunities for TB prevention among patients with TB and DM. This study employed EDTA-treated blood and batch tested it for glycosylated hemoglobin (HbA1c) to test for DM for 333 TB patients. However, the study reported overestimation of prevalence of DM from transient hyperglycemia triggered by TB [46].

Dasa and colleagues in Ethiopia recognised that the cost of screening and treatment was a barrier to patients seeking health services. The cross-sectional study assessed the prevalence and associated factors for depression among TB patients and highlighted the difficulty in covering costs indirectly related to treatment, despite anti-TB drugs being provided for free, which hindered the effectiveness of TB programmes. This was due to the expenses for additional nutritional needs and transportation costs that might result in other knock-on effects such as missed workdays due to fatigue, chest pain, and symptoms of TB, which meant lower earnings and, in turn, heightened psychological distress [47]. Closely related to the concept of financial support, the study conducted in Manila, Philippines by Masumoto and colleagues stated the use of social support scale (Duke-UNC Functional Social Support Questionnaire) being deployed to understand the level of social support received when measuring the depressive states of 561 TB patients [48].

The relatively low number of TB patients needed to be screened for a positive diagnosis such as 24 for hypertension and 5 for proteinuria had been illustrated by Byrne and colleagues as a potentially feasible screening protocol for an integrated TB and NCD model [45]. We acknowledge that the absence of process indicators for evaluating the programme’s feasibility for other NCDs, such as the number needed to screen for MHIs, prevents us from concluding the programme’s feasibility for all NCDs. However, level 1 integration models assist in the early detection of cases otherwise missed. The low cost of implementation, by virtue of using simple and easily deployable screening tools and existing human resources, empowered this integration model [45,47].

The validity of the screening tools used and the probability of socially desirable responses provided by the participants for questionnaire type screening tools were concerns identified by some authors [45,48–50]. Some studies also reiterated other weaknesses of this model such as the lack of confirmatory tools for definitive diagnosis of NCDs after an initial round of positive screening and having heavier workloads on healthcare teams due to additional integrated services could compromise existing TB programmes [45]. However, the authors of 3 studies reported that despite the lack of accuracy of screening tools used, the ease and practicability of screening for multiple NCDs simultaneously proved beneficial for patient management [45,48,51].

Other challenges to the level 1 integration model include the lack of human resources and poor data collection systems for providers, whereas the cost of the added services and logistical barriers (repeated visits) deterred patients from enrolling into the integrated programmes [45,51].

#### Entry via NCDs, testing for TB

There were no studies on an integrated care model with entry via NCDs leading to screening for TB.

### Level 2 integration

#### Entry via TB service, testing and referral for NCD treatment services

Two studies described this integration model where patients entered the system via TB programmes. One study screened for DM and hypertension and the other for DM and MHIs. Both studies included the assessment of risk factors for NCDs and referred the patients for required treatments at appropriate care facilities.

This model was observed in a cross-sectional study by Contreras and colleagues in Peru. Here, TB Cero, a collaborative programme between the Health Ministry and a nongovernmental organisation (NGO) “Socios En Salud” (SES), screened for NCDs such as DM and MHI, and HIV for 192 TB patients. The programme mobilised a field team that functioned using case managers and patient advocates that connected TB patients with appropriate public health facilities for DM and MHI treatment. The costs of treatment were covered by the NGO and TB patients also received social assistance for their unmet basic needs [49].

In the mixed methods study by Anand and colleagues in India, a 2-staged integrated screening for NCDs such as DM and hypertension and their risk factors among 410 TB patients was conducted to assess the yield, feasibility, and acceptability of the programme using process indicators. The patients diagnosed with the DM and/or hypertension were referred to the NCD clinic within the hospital [50].

Manpower and training regarding the screening for NCDs as well as making appropriate referrals were essential for this model to succeed. The collaboration and partnership between the involved healthcare facilities (NCD and TB clinics) are the strengths of this model [49]. Both the patients and the providers appreciated this model of care integration as the added services led to the diagnosis of NCDs otherwise missed [49,50]. Incorporating such services was deemed relevant and important in controlling the TB epidemic as studies have shown comorbidities with NCDs and their risk factors such as low socioeconomic status affect TB treatments and patient outcomes [49,50].

The model by Contreras and colleagues in Peru mentioned above had incorporated an additional yet salient module of managing TB and NCDs, which addressed certain social aspects of patients. This encompassed the provision of food vouchers and home-based treatment support. The lack of social support and basic necessities such as food are known risk factors for TB and NCDs, making this aspect a crucial area that will be delved further in our discussion section [49]. However, the providers indicated the absence of standardised reporting for NCDs services in TB clinics to be a weakness in this integration model. Limitations within the health facilities also affected service delivery as reported by Contreras and colleagues and included the lack of a separate room for NCD screening, which raised privacy concerns among patients and which impeded the uptake of NCD screening services [49].

Other barriers to the level 2 integration model were much like the barriers found in the level 1 integration model, which included long waiting time for screening and referrals, and out-of-pocket (OOP) cost for patients, whereas the challenges for the providers were the additional workload, lack of training to conduct integrated screening services, and shortage of staff and medical supplies for NCD screening.

#### Entry via NCDs, testing and referral for TB treatment services

Three studies elaborated on this model of care integration whereby patients entered the system via NCDs and were subsequently referred to TB clinics for necessary treatment. Patients with DM were screened in 2 studies and 1 study explored the presence of TB in patients with MHIs.

In a study by Zhang and colleagues in China, a specific timeline was allocated for elderly patients with DM from different counties to participate in a free TB screening programme embedded within a health examination exercise conducted at the village level primary care facilities. A total of 93,094 elderly residents from 3 counties participated in the health examinations. The large number of participants enrolling into the programme at the same time overwhelmed the hospital and providers, lowering overall screening quality for TB. This led to a higher possibility of missing out on potential participants requiring the TB treatment service. Many patients also dropped out of the programme and were lost to follow-up due to a weak referral mechanism [52].

A cross-sectional study by Qader and colleagues in Afghanistan noted a 20% higher prevalence of TB among patients with MHIs as compared to the general population. The trained nurses screened 8,073 patients with MHIs for TB during their regular follow-up visits. The patients with presumptive TB were then provided with a sputum test, and all patients with a positive diagnosis of TB were enrolled in nearby direct observation therapy (DOT) centre for further examination and treatment [53].

As this level 2 integration model aims to establish confirmatory diagnosis of TB suspects through further assessments at TB clinics, a more definitive diagnostic tool was not required at the point of entry at NCD clinics. The providers at the NCD clinics could effectively run this integration model after receiving basic training to conduct the TB screening services. This integrated screening and referral model reiterates the impetus for integrated services in settings facing a high prevalence of TB among patients with DM and MHIs [51–53]. The ability to provide opportunistic screening when patients present themselves for NCDs is deemed a key gateway into TB-related interventions, which can help mitigate the spread of TB in LMICs [51,52].

#### Cross-testing and referral for both TB and NCDs treatment services

Three studies describe this model of care integration whereby screening was performed for both TB and NCDs within the same health facility and patients with positive screening results were referred to other care facilities for treatment. Two studies included screening services for DM and 1 study included screening services for multiple NCDs such as DM and hypertension in addition to TB screening services. Two of the studies also screened for HIV, which is of relatively high prevalence in LMICs where the studies were conducted. HIV, however, is not included in our review.

The study by Jerene and colleagues in Ethiopia focused on the lack of the health system’s ability to detect prospective DM cases. This cross-sectional study conducted a tridirectional screening to detect previously missed or undiagnosed TB and DM among 3,439 participants. The study stated that 32.4% of TB patients had abnormal blood sugar suggestive of DM, but the existing health system had detected only 3.5% of these cases, reemphasising the urgency for such integrated screening processes into existing TB or NCD programmes. The same study also highlighted the weakness in its model for TB diagnosis as it was mainly done through symptom screening followed by sputum microscopy. Since most DM patients with TB are asymptomatic, Jerene and colleagues acknowledged the potential underestimation of TB diagnosis among patients with DM [54].

The study by Govindasamy and colleagues in South Africa screened 9,806 participants for TB and HIV and NCDs such as DM and hypertension using a mobile testing unit. The nurses in the mobile testing unit provided the participants that screened positive for TB and/or NCDs with a referral letter to link to healthcare facilities for treatment and follow-up management [55]. Similar to the challenges facing other models, this study also observed a significant loss of continuity of care due to patients losing referral letters or failing to attend follow-up sessions at referred clinics on weekdays due to work commitments. More flexible clinic hours and patient advocacy to improve understanding of the importance of the referral letter and follow-up care were some recommendations provided by the authors [55].

This model of screening and referral for both communicable and NCDs was considered an effective platform for diagnosing TB and NCDs concomitantly, especially in resource-limited settings. The need for such a platform is further emphasised in the cross-sectional study by Araia and colleagues in Eritrea, which points out the shared risk factors such as BMI for the development of TB and DM whereby only 54.5% of the patients in the study were aware that they have both conditions [56]. Furthermore, free screening using mobile units was deemed effective to reach participants in rural areas where screening for diabetes was performed using handheld glucometers and screening for hypertension performed with electronic sphygmomanometers [57]. Provision of trained staff for the integrated screening and referral processes and coordination between the involved healthcare facilities was recognised as a facilitator for the success of this model [57,58].

### Level 3 integration

#### Entry via TB service, colocation of testing and treatment for NCDs

Six studies on this model of integration were found, out of which 5 studies explored the integration of care between TB and various MHIs and 1 study focused on pharmacological care of TB and DM. In this model, the participants entered the system via the TB programme and received care for both TB and NCDs at the same location.

A mixed methods study by Gnanasan and colleagues in Malaysia identified 53 patients with TB and DM comorbidity from their system data and 35 of them that agreed to take part in the study were then provided with a pharmacist-led pharmaceutical care service within the same tertiary hospital to manage their medical conditions. The integrated programme led by pharmacists offered DOT and medication therapy adherence clinical services which were reported to be useful for patients with multiple diseases, namely TB and DM, in managing their medication regiment. This study illustrated that pharmacists could also conduct the integrative services as a form of task sharing. Like other levels of integration, a lack of information flow and coordination was reported. In this study, the disjointed data availability between DM clinics and TB clinics due to the practice of single disease management had made coordination of services across diseases an impediment to integration. In addition, the dilemma of whether to recommend certain monitoring tests or to wait and see if a particular test was ordered by a physician displayed the challenges due to the power dynamics between pharmacists and physicians [59].

Completion of TB treatment can be promoted in programmes with this level of integration as reported in the study by Pasha and colleagues in Pakistan, whereby the integration of an MHI intervention by assimilating integrated practice units (IPUs) into existing TB treatment facilities showed not only improved symptoms of depression and anxiety, but also a significant increase in proportion of TB patients completing their TB treatment with each subsequent MHI intervention session [60]. Additionally, services that include how to manage social stigma and carry positive dialogue regarding mental health conditions with family and friends proved effective in alleviating the effects of depression in patients with TB. The IPU used a task-shifting approach to service delivery, utilising lay health counsellors for the provision of mental health services. The IPUs were designed to embed mental health screening and counselling services into the existing treatment flow at each facility, so as to not affect normal TB related operations in the facilities [60].

Resources such as trained health professionals and medical supplies are essential for providing the added care in this integration model. For example, a nurse and 2 medical students were trained for the integrated programme as reported in a study by De Castro-Silva and colleagues in Brazil [61], and a designated team of pharmacists and physicians worked together to design an individualised pharmaceutical care plan for TB and DM in the study conducted in Malaysia by Gnanasan and colleagues [59]. De Castro-Silva and colleagues had further reported that certain tools such as the Patient Health Questionnaire-9 (PHQ-9), which was originally a self-administered questionnaire had required providers to administer due to the population’s low literacy rate, thus showing the need to train providers to offer tailored services based on requirements of the target population.

A mixed methods study by Walker and colleagues in Nepal had involved the services of an NGO. This study screened 135 patients receiving MDR-TB treatment for MHIs. This integration model enabled Nepal’s National Tuberculosis Programme (NTP) to identify and develop contextually relevant psychosocial interventions that could be delivered within routine NTP services. The services administered were in a stepped-care format. The first step was to provide information and educational materials pertaining to MDR-TB and its treatment options to patients and their family followed by a screening process for NCDs during follow-up monthly visits. Subsequently, brief counselling sessions for overall health based on behavioural activation psychological therapy, group support for selected patients to reduce their negative emotions, and provision of social support for eligible patients were also provide in this integration model. An NGO that delivered mental health services also trained providers to provide group counselling services to patients. However, the authors reported that group counselling was not suitable for all patients as some faced difficulties expressing themselves in a group setting, while others became more worried after hearing the problems faced by fellow patients. Language barriers during counselling sessions also surfaced. The infrastructural limitation due to the lack of an appropriate, private space to conduct the counselling at the centre (which was also the case with screening) often created distractions for the patients and counsellors during the counselling sessions. The counsellors had to improvise and used rooms that were shared with other services or an outside space that provided more privacy to conduct the counselling sessions. Despite the difficulties mentioned, patients had expressed the benefits of hearing experiences of a cured patient during the group counselling sessions [58].

The mixed methods study by Lovero and colleagues in South Africa evaluated the integration of mental healthcare into TB and maternal–child healthcare services in 4 different districts involving 40 clinics (10 per district) [57]. A stepped-care approach was employed where patients were screened for MHIs and treated at the same clinic or referred to specialised care based on severity of the condition. Additionally, after the patient’s condition was stabilised, a down-referral back to the original primary care clinic was performed. In-depth interviews were conducted with 9 district-level programme managers (DPMs), 17 mental health practitioners (MPHs), and 59 nurses to understand the feasibility and barriers of the programme. This study homed in on more meso and macro-level barriers such as the absence of coordination across health programmes in district-level administration, lack of material and human resources, and low mental health awareness in both the district administration and general population. Low mental health literacy in both the district administration and general population made mental health a low priority for integration into other existing programmes. Providers also highlighted that poor coordination within and across governing bodies and limited understanding of the need for integrated care at the administrative and governance level were weaknesses of this integration model. Therefore, despite TB and NCD services being colocated or within close physical proximity, providers highlighted that poor interprogramme coordination and communication plus conflicting directives from different programmes were areas that required improvement [57].

#### Entry via NCDs, colocation testing and treatment for TB

There were no studies on an integrated care model with entry into the system via NCDs and colocation for treatment.

### Facilitators and barriers to integration of TB and NCD services

Salient and overarching themes regarding facilitators and barriers facing integrated service delivery models were mapped to the WHO health systems framework which comprises six dimensions are summarised in Table 7 below.

**Table 7:** Summary of facilitators and barriers mapped into WHO health system framework.

Health system building blocks	Barriers	Facilitators
Service delivery	• Cultural beliefs and preconceptions about disease conditions posed huge hurdles at the personal level • Poor public awareness of TB- and NCD-related comorbidities led to poor uptake • Absence of monitoring parameters • Lack of a conducive space to conduct screening and/or counselling services due to privacy concerns of patients • Prolonged waiting times at clinics and also extended intervals before receiving referral care at onwards care facilities • Gender of the provider might be a concern for particular groups of patients especially when taking anthropometric measurements • Missed screenings due to undefined roles resulting in providers being unclear of job scope and responsibility in the cascade of integrated care	• Colocation of screening and treatment services for both TB and NCDs increased physical accessibility for patients, particularly if patients were already visiting the care facilities for either service • Screening processes were smooth, and the questions asked during screening were simple, understandable, and palatable • Community health workers were engaged to deliver medicines to patients place of residence when required • Provision of care at patients’ home achieved through mobile clinics/units reduced physical barriers to uptake of services
Human resources	• Limited human resources for screening, treatment and referral services • Increased workload for the providers already overstretched by their current workflows • Limited/inadequate training provided to providers to conduct the additional screening /treatment services • Providers were overworked and confused due to directives from multiple programmes that had separate health agendas	• Motivated providers: goes the extra mile to provide for the patients and were willing to work with the resources (although might be limited) that were made available • Task shifting was promoted in some programmes whereby nonmedically trained personnel were empowered to conduct the screening and referral services
Medical products and vaccine	• Lack of medication or potential stockouts • Lack of medication adherence due to prolong management period • Deployment of suboptimal screening tools due to operational circumstances such as cost concerns and for simplicity purposes, at the expense of screening accuracy	
Sustainable financing and social protection	• OOP expenditure for the patients might deter uptake of services due to financial barriers • No additional budget provided to providers to conduct the additional services for screening or treatment	• Usage of inexpensive yet moderately accurate screening tools • Screening/treatment for some integrated programmes was provided at no cost or reduced cost to patients
Information	• Lack of structured and standardised reporting formats for services carried out in the programme within the same or different facility led to difficulty in standardising the tracking of patient parameters and services delivered • Poor referral system led to loss to follow-up • Lack of a shared electronic medical record system results in poor coordination and communication between different care providers within or across facilities	
Leadership and governance	• Lack of national guidelines for TB/NCD care management and integrated service delivery frameworks • Lack of collaboration between providers within and beyond the health system resulted in disjointed agendas • Lack of political commitment resulting in inadequate allotment of funds to sustain integrated programmes	• Government’s willingness to partner nonprofit/NGOs to complement existing government-run programmes

NCD, noncommunicable disease; NGO, nongovernmental organisation; OOP, out-of-pocket; TB, tuberculosis; WHO, World Health Organization.

## Discussion

This review identified the potential applicability of integrating TB and NCD services in LMICs. Using the data extracted from the included studies, we derived key elements of the different levels of integration for TB and NCD services, followed by categorising them into 3 distinct levels, differentiated according to entry points into the health system. Our findings suggest that higher levels of integration conferred more benefits to patients in terms of managing TB and NCDs. However, we are also cognizant that numerous aspects spanning the 3 levels of integration are overlapping yet differ in certain operational areas depending on the study setting. Due to the pluralistic approaches taken by integration models that vary from how they exploit opportunistic screenings at either point of disease entry to onwards care treatment, the application of a complex systems approach to integrated services planning, delivery, and evaluation is, therefore, central to deciphering intervention implementation and impacts in real-world settings. We accomplished this by mapping the operational features of the various models of integration to WHO health systems framework, which provided a validated structure to organise our findings. As such, we elucidated the overarching facilitators and barriers that fall within the dimensions of service delivery, human resources, sustainable financing and social protection, information, and leadership and governance.

Our findings suggest that care integration is more common with entry via TB to the system, with 19 studies supporting this. Eight studies were conducted at level 1 integration, 8 studies at level 2 integration, and 6 at level 3 integration. A higher level of integration that requires longer chains of care as compared to a lower level of integration, whereby less services or services catering to one disease only is provided, comes with increasing challenges that need to be iteratively surmounted at all levels of the health system. More integrated models need to be mindful that additional integrated services do not compromise the functionality of existing TB or NCD programmes or clinical operations.

Integration aims to address fragmentation in services, enabling better coordinated and more continuous care. In short, integration is achieved by careful planning and financing, with a shared vision centred on a target patient population [62]. All studies pointed to integration as the genesis of structural links between previously separate services, encompassing aspects from organisational modifications, resource planning to physical colocation. At the lowest level of integration, only screening for either TB or NCDs is provided. In most studies, low-cost and straightforward screening techniques for TB and NCDs are used by trained staff. Level 1 integration studies had illuminated that providing point-of-care screening by either disease entry point, but without receiving the needed treatment through onwards referral or in-house treatment is insufficient to mitigate the increase in prevalence of either TB or NCDs effectively. The lack of providing referral to onwards care postscreening could be attributable to reasons such as the lack of comprehensive operational planning since such integrated programmes are relatively new in LMIC settings and cost concerns whereby the funds allocated for such programmes might have limited the prospects of linked care which may be considered unethical and a wasteful expenditure of scarce resources for diagnostic tests without any impact on patient management or outcomes.

Therefore, level 2 integration is more optimal in that regard but has its own challenges. Despite having referrals made to appropriate providers for both TB and NCD treatment postscreening, level 2 integration models face new difficulties that the level 1 integration models did not operationally face. This includes lost to follow-up due to loss of referral documents and patients facing the inconvenience of attending another visit, which might be constrained by work commitments and physical distance to the referred clinic [55].

Hence, full integration at level 3, whereby screening and treatment occur at the same location, has been shown in our reviewed studies to provide the best point of care for both disease groups and promotes, to an extent, care integration and continuity of care. Several studies have shown that colocation of services derives better health outcomes, follow-up care adherence, and overall higher patient satisfaction [63,64]. High levels of integration have also been shown to reduce fragmentation of care, thus minimising resource wastage and inefficiency by avoiding duplication of services and contradictory decisions by providers [65]. However, as direct outcome measures were not reported in the reviewed studies, we can only surmise that having the deepest levels of service integration confer the most benefits to both patients and the health system.

TB and NCDs control programmes must touch every aspect of a nation’s health system to manage the diseases comprehensively. Herein, we will discuss the facilitators and barriers with the health systems components in mind to further unpack the operational features of a high functioning integration model embedded in the health system of LMICs. Notably, 2 studies highlighted the need to factor in the social and financial elements for integrated models of care. Both factors fall beyond the remit of traditional TB control programmes. As sustainable financing and social protection is one of the dimensions of WHO health systems framework, we will use this as the entry point to delve deeper into other health systems dimensions using the facilitators and barriers summarised in Table 7 above.

First, sustainable financing and social protection is central to the management of TB and NCD integrated programmes as TB and NCDs have close links to poverty and social activation [66]. Hence, integration models will require more from health systems than the traditional medically oriented interventions that do not focus on the financial and social aspects of patients. OOP payment is shown to reduce the uptake of essential health services, where patients in LMICs might prioritise nonmedical yet essential aspects of their lives such as the purchasing of food and shelter. This will, in turn, become a missed opportunity if costs are the limiting factor to the uptake of TB and NCDs services. Therefore, health systems must explore robust financing and social protection mechanisms to promote the uptake of services at all levels of integration to ensure maximum reach and impact of integrated programmes [67]. From the providers and policymakers point of departure, the terms universal health coverage (UHC) and limited fiscal space lie in a luminal space, whereby finite resources need to be allocated to various aspects of the health system [68]. The study by Contreras and colleagues conducted in Peru showed that meeting the high demands for screening services necessitated the engagement of private laboratories where the costs were covered by the programme, while socioeconomic assistance such as transportation reimbursement and food vouchers were rendered to the patients by an NGO [49]. Therefore, having TB and NCD services provided at one facility can potentially reduce transport costs due to multiple visits. However, we are unclear of how the medical costs incurred by the patients will be divided in an integrated programme since certain medical conditions such as TB might already be covered by an existing programme but not others.

More importantly, risk factors for TB and NCDs are often multifaceted, and the most cost-effective way to mitigate both epidemics might be through early detection. Thus, financial attributes of screening services must consider the socioeconomic status of the targeted population and the ability of the country to finance it sustainably. This was further emphasised in another study by Byrne and colleagues in Peru, whereby glycosuria was used as a biomarker for undiagnosed DM and inadequate glycemic control for DM and CKD patients. Despite having moderate sensitivity, the study failed to employ more specific assessment tools for renal function impairment such as serum creatinine, due to cost and implementation feasibility concerns [45].

Second, at the service delivery level, contextual nuances such as cultural beliefs and preconceptions about a disease condition pose huge hurdles at the personal level and also impede the full functionality of an integrated programme. Poor or lack of public education and awareness of TB and NCDs can create misconceptions about the diseases such as “leaving it to fate.” This holds true not only for patients but also for policymakers, as seen in the disconnect from TB and NCDs agendas mentioned in the study by Lovero and colleagues [57]. A facilitator that can be employed to overcome this is to increase health literacy through community health workers [69]. The deployment of validated guidelines ensures that evidence-based process and outcome indicators are being monitored and fulfilled as cornerstones for effective patient-centred care, especially for TB and NCDs, which require prolonged management [70]. Furthermore, accessibility to care can also pose a barrier due to transportation costs or heightened opportunity costs for informal labourers when attending follow-up services. These challenges can be surmounted by deploying mobile testing units as used in the study by Govindasamy and colleagues in South Africa to meet patients where they are [55]. The lack of conducive space to screen for NCDs and provide counselling services might cause privacy concerns. As a result, patients might be less willing to take up these additional services, be it for mental health treatment or screening for other NCDs where anthropometric measurements are taken. Although not reported in the studies reviewed, colocation with TB services necessitates extra infectious disease precautions to reduce the nosocomial spread of TB when non-TB patients present themselves for NCDs [71].

Third, at the human resources level, it was apparent in nearly all reviewed studies that the increased workload posed a considerable roadblock to the operations of integrated programmes. This is especially salient in LMIC settings whereby training of health workers typically involve only a particular disease group such as maternal and child health or specific infectious diseases, while cross-training (i.e., TB and NCD services) for more than 1 disease condition is rare [72]. Additionally, the lack of manpower and training or expanded job scope places more stress on existing providers, who are already stretched by their normal clinical operations [73]. The overextending of these providers might lead to screening misses and treatment errors. As such, providers must not only be well trained but also motivated to provide the services. Attitude, professionalism, and commitment create stronger patient–provider relationships, which confer better patient outcomes and, hence, essential elements to any integration model [74].

Fourth, the availability of medical products and vaccines is essential to the implementation of integration programmes. However, aside from Bacillus Calmette–Guérin (BCG) vaccine, whose effect wanes after childhood, there are effectively no viable vaccines for TB. NCDs are also primarily developed due to genetic or lifestyle predisposition, which can only be prevented through behavioural modifications and medications [75]. Thus, the availability of efficacious medicines for TB, administered mainly through DOT, is an integral element to any TB programme. A break in the medication regime or shortage of medicines at all levels of integration can lead to the generation of MDR-TB [76]. Likewise, the provision of high-quality medication for NCDs is required to ensure optimal management of NCDs, which are often prolonged and require extended medication regimes and monitoring. Additionally, there is also a need for medical resources that aid in screening of patients for TB and NCDs. For example, the availability of handheld glucometers used for screening DM and electronic sphygmomanometers for hypertension as explicated by Govindasamy and colleagues provided a practical way to screen patients for NCDs [55]. Without the provision of medical equipment for screening, linkage to onwards care and treatment will be rendered ineffective in an integrated service delivery model. Although not discussed in the articles reviewed, having integration models that are equipped with adequate medical resources for both TB and NCDs is essential to managing both disease conditions concurrently. This is because the inability to control one might lead to the exacerbation of the other. For example, uncontrolled DM is associated with worsening of TB control as these 2 conditions are closely associated biologically [19].

Fifth, the information provided within and across healthcare entities is an essential tenet for the smooth running of any health programme, let alone one that integrates services catering to multiple disease conditions across different providers. Many of the studies reviewed highlighted the lack of coordination across providers, especially across different healthcare institutions, which hindered integration on many fronts. There is often a lack of common electronic medical record system or standardised medical information storage platform that tracks process and clinical outcome parameters of the patients and data access to providers within and across echelons of care was also reported to be limited. For example, Gnanasan and colleagues had expressed that certain clinical parameters such as blood pressure were not checked at the TB clinic but could have been extracted from DM or cardiovascular clinic’s records instead in order to give providers a more comprehensive medical history of the patient. However, the lack of coordination across provider settings stymied the exchange of patient data [59]. Furthermore, the information tracked must be evidence informed and relevant for clinical management of the patients’ condition. To this end, validated guidance principles and reporting measures/frameworks need to be deployed by providers. However, many reviewed studies had reported the lack of a former reporting system. Many had to turn to operationalised indicators to track the patient pathway, which might not be optimal. An example is using a modified WHO STEPwise Approach to NCD Risk Factor Surveillance (STEPS) framework that Anand and colleagues reported [50]. Although only pilot tested once in the study’s context, the operationalised questionnaire that screens for NCD predisposition was deemed quick and easy by both patients and providers. Therefore, despite the absence of thorough validation, rapid screening procedures can be deployed in resource constraint settings to streamline protocols to refer patients for onwards care.

Last, leadership and governance are central to implementing any integrated programme vis-à-vis other core functions that ensure the programme’s sustainability and credibility within a health system. Governance also cuts across all other aforementioned health system functions, making transparent, accountable and committed stewardship paramount in TB and NCD control. Good governance will also require taking the lead in upholding newly minted partnerships that include formal and informal productive relationships that span within and beyond the health sector and between the health system, its intermediaries and end users (patient and providers) through the accountable provision of evidence-driven guidelines and adequate and responsible incentivisation. An example of active collaboration is displayed in the study by Contreras and colleagues whereby the TB Cero programme was implemented as an integrative partnership model between public hospitals and an NGO in Peru [49]. At the national level, having a platform to bring together intersectoral actions driven by strong political commitment coupled with a definition of integration that captures an explicit agenda and a clear roadmap for implementation undergirds organisational change towards more assimilated TB and NCD service models. Specifically, governments can also promulgate national TB and NCD plans/guidelines/frameworks and earmark the needed funds to run plans into fruition. This was alluded to in the study by Ekeke and colleagues in Nigeria, whereby patients with DM were screened for TB, in line with the national TB guidelines [51]. Thus, there is a need to update these guidelines to include NCDs to prevent any missed opportunities in identifying NCD cases. Lönnroth and colleagues had also proposed a TB elimination framework to draw key takeaways from prospective TB and NCD integration models. This encompasses universal access to high-quality TB and NCD service with a lens that places groups at the highest risk first and reducing underlying vulnerabilities by incorporating health lenses in all policies, bolstering long-term political will for locally spearheaded integrated programmes, and garnering cross-sectorial and multilevel buy-in for the implementation of global TB and NCD action plans [77]. Finally, governance bodies must be mindful of the interconnectedness of interventions along the “cascade of care” from detection of TB or NCDs to successful completion of treatment or long-term management. This implies that governance bodies must be able to evaluate programmes using both process and clinical outcome measurements in a joined-up rather than a standalone manner to truly ascertain the effectiveness of the integration of TB and NCD services within one programme and at a common locale. As the effectiveness of the integrated programmes go beyond a single entity and across different sectors outside the health system, the need to incorporate systems thinking concepts as well as adopting relevant disciplines such as social sciences and health policy analysis will serve as cornerstones for a joined-up systems approach to evaluation [78].

This systematic review foregrounds the gaps in both policy and research in the areas of TB and NCD service integration. Policymakers and researchers have focused mainly on integrating TB and HIV services, as seen from the various existing and well-evaluated programmes and systematic reviews performed [33,37,79–82].

The research gaps can be plugged with more monitoring and evaluation works that track the process and clinical outcomes through validated indicators using a prospective or retrospective cohort study, case–control, or randomised control study approaches. Furthermore, the experiences and expectations of patients and providers can be explored through qualitative studies to confer appropriate models of care integration that meet the needs of all stakeholders. The urgency is now more than ever in the face of a swelling TB and NCD burden, and prospective research findings must be able to empower policymakers to drive integration models using implementation science and sustained political will.

Policymakers and programme implementers must recognise that the inability to attain sustainable and effective integration might be less attributed to ineffective programmes per se but rather due to the impediments underlying the health systems building blocks. Hence, integration necessitates stakeholders to envision the channelising of resources and agendas beyond individual programmes and to an extent sectors, while concomitantly investing in all health systems building blocks.

Although there is no magic bullet to achieve a fully integrated configuration for all settings, some actionable steps surfaced. We have summarised the key facilitators to be accounted for and barriers to be avoided during the implementation of integration models in Table 7 above. The high start-up costs to initiate such programmes require policymakers to place long-term returns at the forefront when deciding to allot resources into these models as return on investment in terms of better population health outcomes can only be seen in the longer term. Spillover effects such as better infrastructure and trained manpower can also enhance the health sector and improve access to care for the general population. Uptake must also be sustainable through carefully tailored integration models that account for the needs and preferences of the local population. A salient point that did not appear from our review but is crucial for the uptake of integrated services is the reduction of stigma when seeking care for certain services associated with TB and MHIs, which might affect the extent to which such models of integration are palatable.

This systematic review was limited by the dearth of research exploring the experiences of patients and providers, and specific process indicators that evaluate the feasibility of integrated interventions, as most studies focused on reporting outcomes at a particular time period. Furthermore, the main bibliographic databases used in this review may have had a limited coverage of material from LMICs regarding TB and NCD integrated programmes, and we acknowledge that the databases might have lacked coverage of material not published English [83–85]. In addition, the heterogeneity of the study designs, contexts of study, participants, sample sizes, outcomes, interventions, and diseases studied can potentially limit the generalisability of the study findings. Importantly, this review also revealed that most integration models were contextually bound, with each LMIC having their very own set of healthcare and political agendas writ large. Therefore, our findings have extracted the overarching themes that are cross-cutting and salient to LMICs, particularly in Asia, Africa, and the Americas.

One strength of this review is the usage of a widely deployed health system framework to guide the data analysis and reorganisation of themes for the facilitators and barriers, reducing the variability across research team members. However, we must be cognizant of the oversimplification of intricate process pathways and organisational culture. In practice, many of the key issues span across multiple building blocks, as to be anticipated when employing a health systems lens. However, we managed to utilise this framework for mapping integrated service options to each health system building block to derive high-quality and pragmatic design options for integration elaborated above.

Another strength is the development of a new framework that categorises models of integration according to their depth of integration. This will enable both policymakers and researchers to understand where a country’s TB and NCD programmes lie on the spectrum of integration and, with that knowledge, strive towards the deepest levels of integration by avoiding the barriers and incorporating the facilitators into their programmes.

## Conclusions

The burgeoning number of TB and NCD cases has made a strong case for tackling this combined burden of TB and NCD prevalence with a comprehensive and integrated approach commensurate to its disease burden. Limitations notwithstanding, this systematic review provides valuable insights into integrating TB- and NCD-related services into one common programme in LMICs. This has the potential to improve health service delivery across disease conditions and levels of care that can lead to population health benefits.

## Supporting information

S1 Checklist PRISMA 2020 for Abstracts Checklist. PRISMA, Preferred Reporting Items for Systematic Reviews and Meta-Analyses.
(DOCX)

S2 Checklist PRISMA 2020 Checklist. PRISMA, Preferred Reporting Items for Systematic Reviews and Meta-Analyses.
(DOCX)

## Abbreviations

BCG: Bacillus Calmette–Guérin
CKD: chronic kidney disease
CVD: cardiovascular disease
DM: diabetes mellitus
DPM: district-level programme manager
DOT: direct observation therapy
HbA1c: hemoglobin
IPU: integrated practice unit
JBI: Joanna Briggs Institute
LMIC: low- and middle-income country
MDR-TB: multidrug-resistant tuberculosis
MHI: mental health illness
MHP: mental health practitioner
NCD: noncommunicable disease
NGO: nongovernmental organisation
NTP: National Tuberculosis Programme
OOP: out-of-pocket
PHQ-9: Patient Health Questionnaire-9
PRISMA: Preferred Reporting Items for Systematic Reviews and Meta-Analyses
SES: Socios En Salud
SIGN: Scottish Intercollegiate Guidelines Network
STEPS: STEPwise Approach to NCD Risk Factor Surveillance
TB: tuberculosis
UHC: universal health coverage
WHO: World Health Organization
